# Gα13 negatively controls osteoclastogenesis through inhibition of the Akt-GSK3β-NFATc1 signalling pathway

**DOI:** 10.1038/ncomms13700

**Published:** 2017-01-19

**Authors:** Mengrui Wu, Wei Chen, Yun Lu, Guochun Zhu, Liang Hao, Yi-Ping Li

**Affiliations:** 1 Department of Pathology, University of Alabama at Birmingham, SHEL 810, 1825 University Boulevard, Birmingham, Alabama 35294-2182, USA

## Abstract

Many positive signalling pathways of osteoclastogenesis have been characterized, but negative signalling pathways are less well studied. Here we show by microarray and RNAi that guanine nucleotide-binding protein subunit α13 (Gα13) is a negative regulator of osteoclastogenesis. Osteoclast-lineage-specific *Gna13* conditional knockout mice have a severe osteoporosis phenotype. *Gna13*-deficiency triggers a drastic increase in both osteoclast number and activity (hyper-activation), mechanistically through decreased RhoA activity and enhanced Akt/GSK3β/NFATc1 signalling. Consistently, Akt inhibition or RhoA activation rescues hyper-activation of *Gna13*-deficient osteoclasts, and RhoA inhibition mimics the osteoclast hyperactivation resulting from *Gna13*-deficiency. Notably, Gα13 gain-of-function inhibits Akt activation and osteoclastogenesis, and protects mice from pathological bone loss in disease models. Collectively, we reveal that Gα13 is a master endogenous negative switch for osteoclastogenesis through regulation of the RhoA/Akt/GSK3β/NFATc1 signalling pathway, and that manipulating Gα13 activity might be a therapeutic strategy for bone diseases.

## Abstract (web summary)

Osteoclasts are bone resorptive cells and an attractive target to treat diseases characterized by excessive bone loss, but little is known about osteoclast inhibition. Here the authors identify Gα13 as an intracellular inhibitor of osteoclastogenesis that can prevent bone loss in mice via Akt activation and inhibition of RhoA signalling.

Osteoclasts are the principal, if not exclusive, bone-resorbing cells^1^,^2^,^3^. A balance between bone formation by osteoblasts and bone resorption by osteoclasts is critical to maintain normal bone density and mineral homeostasis^1^,^2^,^3^. Osteoclastogenesis is initiated by signals transmitted by the receptor activator of nuclear factor-κB (RANK) ligand (RANKL) and macrophage colony-stimulating factor (M-CSF)^1^,^2^,^3^,^4^. RANKL and M-CSF, binding to their receptors, RANK and cFms, respectively, on the surface of osteoclast precursors, activates many key transcription factors including nuclear factor of activated T-cells, c1 (NFATc1) and CCAAT/enhancer-binding protein alpha (C/EBPα)^1^,^2^,^3^,^4^. Our previous work on cathepsin K^5^,^6^, Atp6i (refs 7, 8), C/EBPα^9^ and RGS10 (ref. 10) has contributed to the discovery of positive regulatory machinery controlling osteoclast differentiation and function. Notably, excessive osteoclast activity is responsible for various bone diseases including osteoporosis, rheumatoid arthritis and Paget's disease^1^,^2^,^3^. Thus, we anticipate that hyperactivation of osteoclasts should be antagonized by intrinsic negative regulators, which have received limited attention, to maintain normal bone homeostasis. A decrease in the expression of negative osteoclast regulators might trigger the bone loss associated with many pathological bone disorders.

Guanine nucleotide-binding protein subunit α13 (Gα13; encoded by *Gna13*) belongs to the G12 subfamily of the G protein superfamily. Gα13 regulates cell cytoskeleton organization by regulating the RhoGEF-RhoGTPase signalling pathway^11^,^12^. Here we utilize a genome-wide screening strategy and characterize Gα13 as an intrinsic negative regulator of osteoclast formation and activity. Absence of Gα13 favours osteoclast formation and enlarges osteoclast size, leading to an osteopenia phenotype in mice. On the other hand, constitutively active Gα13 blocks the formation of multinucleated osteoclasts and their bone resorptive activity. We reveal that Akt-mediated signalling, regulated by RhoA, is critical for the function of Gα13 in osteoclasts.

## Results

### *Gna13* silencing promotes osteoclastogenesis

**Figure 1:**
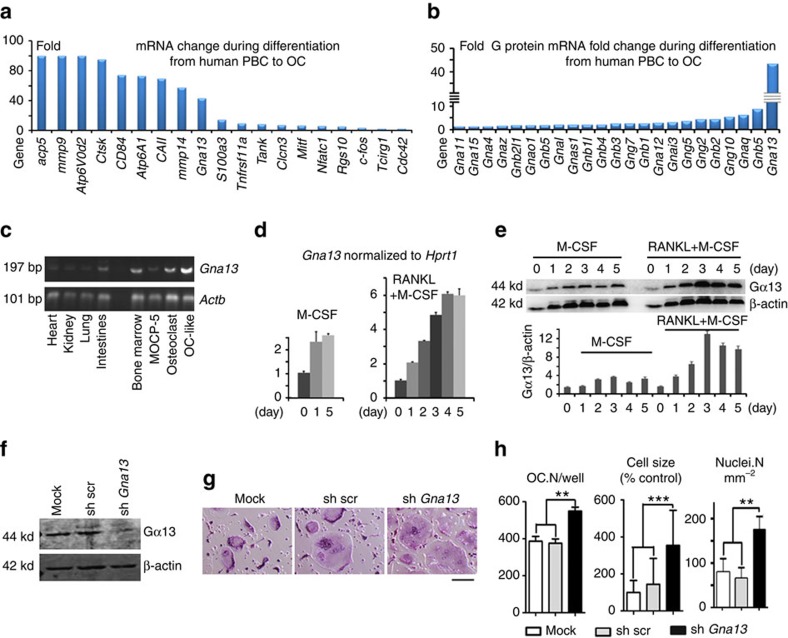
Gα13 which is induced by RANKL and M-CSF inhibits osteoclastogenesis. (**a**,**b**) Microarray profile of gene expression during osteoclast differentiation. OC, osteoclasts; PBM, peripheral blood monocytes. (**c**) Semi-quantitative reverse transcription–PCR to detect *Gna13* mRNA level in murine tissues and cells. (**d**) Quantitative reverse transcription–PCR to analyse time-course expression of *Gna13*, normalized to *Hprt1*, in murine bone marrow monocytes (BMMs) under the treatment of M-CSF, or M-CSF and RANKL; *N*≥4. (**e**) Western blot to analyse time-course expression of Gα13 in BMMs under the treatment of M-CSF or M-CSF and RANKL, and its quantification in lower panel; *N*≥4. (**f**) Western blot to analyse Gα13 expression in non-infected BMMs (mock) and BMMs infected with lentivirus expressing scrambled short hairpin RNA (shRNA) (sh-src) or shRNA targeting Gna13 (sh-*Gna13*). (**g**) TRAP staining to detect osteoclast formation of mock, sh-src and sh-*Gna13* BMMs. (**h**) Quantification of cell number (OC.N/well and nuclei.N mm^−2^; *N*≥10) and relative cell size (*N*≥100) in **g**. OC.N/well, osteoclast (TRAP positive multinucleated cell) number per well. Nuclei.N mm^−2^, osteoclast nuclei number per mm^2^. Results in **d**,**e**,**h** are expressed as mean±s.d.; **P≤0.01; ***P≤0.001 (Student's *t*-test). Scale bars, 200 μm.

Given the limited attention attributed to the investigation of negative regulators of osteoclastogenesis, we performed a genome-wide screening to identify factors that negatively regulate osteoclast formation (Fig. 1). Towards this end, we compared gene expression in human blood monocyte-derived osteoclasts with that in their precursors, and found that *Gna13* was not only drastically induced by RANKL, but also its expression pattern was comparable to various osteoclast genes (Fig. 1a), and much higher than the genes encoding several other G proteins (Fig. 1b). Moreover, gene expression analysis confirmed that *Gna13* was highly expressed in osteoclasts and osteoclast-like cells derived from MOCP-5, an osteoclast precursor cell line that was generated in our lab^13^, as compared with several tissues, such as heart, kidney, lung and intestine (Fig. 1c). Furthermore, using murine bone marrow monocytes (BMMs), which are widely used as primary osteoclast precursors, we demonstrated that whereas Gα13 expression was only mildly induced by M-CSF (∼2-fold at both mRNA and protein level), its expression was strongly induced by combined stimulation with M-CSF and RANKL (∼6-fold at mRNA level and 8-fold at protein level) (Fig. 1d,e). These results indicated that Gα13 might have important roles in osteoclasts. Hence, we then silenced Gα13 expression by lentiviral-mediated expression of short hairpin RNA against *Gna13* in BMMs. The knockdown efficiency (∼90%) was confirmed by immunobloting as compared with control cells (Fig. 1f). Interestingly, our data showed that *Gα13* silencing strongly increased osteoclast formation and also drastically enhanced osteoclast size (Fig. 1g,h). These results suggested that Gα13 might be a negative regulator of bone resorption.

### Depletion of *Gna13* causes osteopenia in mice

**Figure 2:**
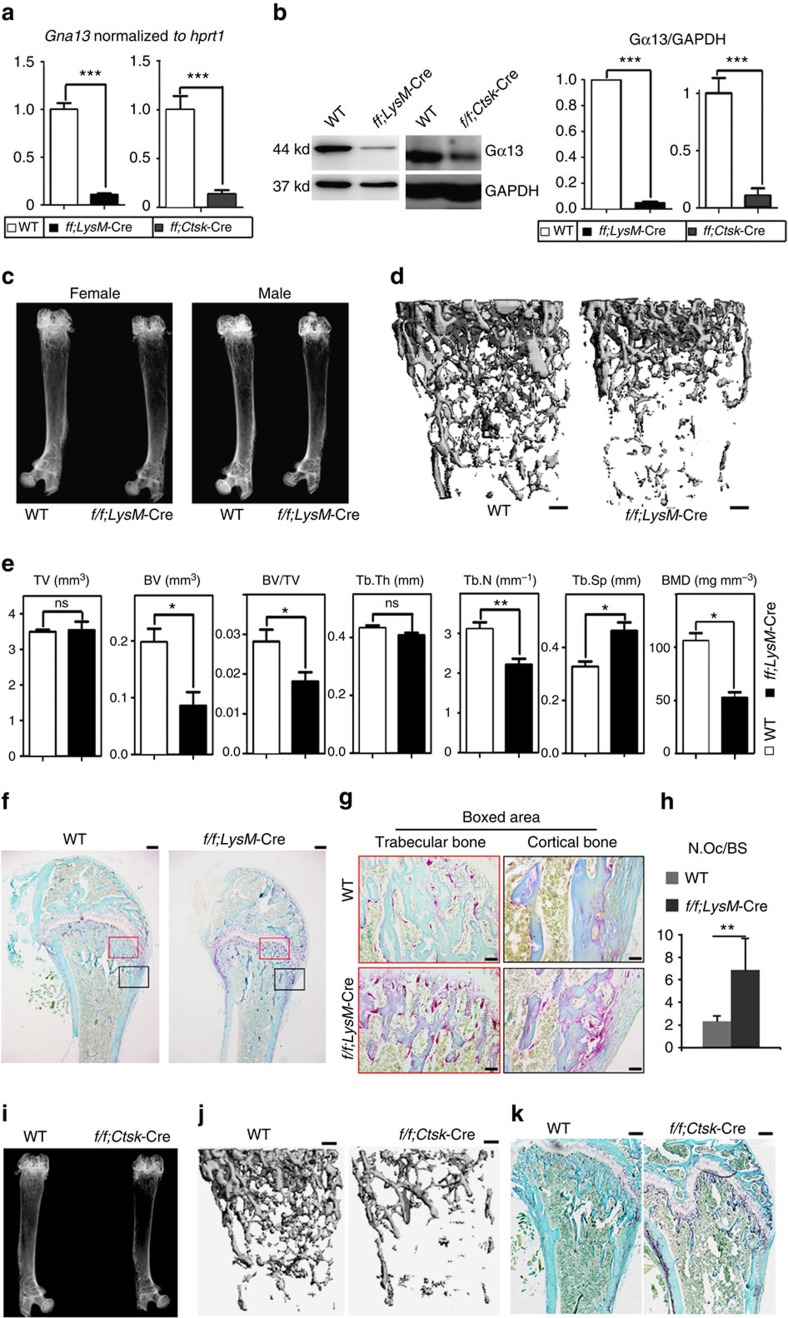
Osteoclast-specific *Gna13*-CKO mice displayed osteoporosis. (**a**) Quantitative reverse transcription–PCR confirmed deletion of *Gna13* mRNA in day-1 *Gna13*^*f/f*^*LysM*-Cre (*f/f;LysM*-Cre) and day-4 *Gna13*^*f/f*^*Ctsk*-Cre (*f/f;Ctsk*-Cre) pre-osteoclasts; *N*=6. (**b**) Western blot confirmed deletion of Gα13 protein in *Gna13*^*f/f*^*LysM*-Cre BMMs and day-5 *Gna13*^*f/f*^*Ctsk*-Cre pre-osteoclasts. Quantification is co-presented in the right panels; *N*=6. (**c**) Radiographs of two-month-old wild type (WT) and *Gna13*^*f/f*^*LysM*-Cre male and female mice femurs. (**d**) μ-CT analysis of two-month-old WT and *Gna13*^*f/f*^*LysM*-Cre female mice femurs. (**e**) Quantification from **d**; *N*=6. BMD, bone mineral density; BV, bone volume; Tb.Th, trabecular bone thickness; Tb.N, trabecular bone number; Tb.Sp, trabecular bone space; TV, tissue volume. (**f**) TRAP staining of 2-month-old WT and *Gna13*^*f/f*^*LysM*-Cre female mice femurs. (**g**) High magnification images of **f**. (**h**) Quantification of N.Oc/BS (osteoclast number / bone surface) in **f**; *N*=6. (**i**) Radiographs of two-month-old WT and *Gna13*^*f/f*^*Ctsk*-Cre female mice femurs. (**j**) μ-CT analysis of two-month-old WT and *Gna13*^*f/f*^*Ctsk*-Cre female mice femurs. (**k**) TRAP staining of 2-month-old WT and *Gna13*^*f/f*^*Ctsk*-Cre female mice femurs. WT, wild-type. Results were expressed as mean±s.d.; *P≤0.05; **P≤0.01; ***P≤0.001; ns, not significant (Student's *t*-test). Scale bars in **d**,**f**,**j**,**k**, 200 μm; Scale bar in **g**, 20 μm.

To further investigate the role of Gα13 in osteoclast formation and activity, we generated knock-out mice through specific deletion of *Gna13* in the osteoclast lineage (Fig. 2). Mice bearing *loxP* sites encompassing the *Gna13* exon2 (*Gna13*^*f/f*^ mice)^14^ were crossed with those expressing Cre recombinase driven by the *lysozyme M* promoter (*LysM*-Cre mice) or the *Cathepsin K* promoter (*Ctsk*-Cre mice). The offspring were intercrossed to get *Gna13*^*f/f*^*LysM*-Cre mice or *Gna13*^*f/f*^*Ctsk*-Cre mice and wild-type (WT) mice. Mouse genotypes were confirmed by PCR (Supplementary Fig. 1a–c). The deletion of Gα13 in *Gna13*^*f/f*^*LysM*-Cre BMMs and *Gna13*^*f/f*^*Ctsk*-Cre pre-osteoclasts was confirmed by quantitative PCR and immunoblotting (Fig. 2a,b). While *LysM*-Cre deletes *Gna13* in the osteoclast precursors (Fig. 2a,b), *Ctsk*-Cre works at a late stage of osteoclast differentiation (Supplementary Fig. 1d).

Two-month-old WT and *Gna13*^*f/f*^*LysM*-Cre or *Gna13*^*f/f*^*Ctsk*-Cre mice have no obvious gross morphological changes. As assessed by X-ray analysis, skeletal mass was decreased in distal femurs of both male and female *Gna13*^*f/f*^*LysM*-Cre mice as compared with those of WT littermates (Fig. 2c). Quantitative microtomography (μ-CT) analysis showed that *Gna13*^*f/f*^*LysM*-Cre mice exhibited ∼30% reduction in bone volume/tissue volume (BV/TV) and trabecular number (Tb.N) as well as ∼40% increase in trabecular space (Tb.Sp), ∼50% decreased in bone mineral density (BMD) as compared with WT littermates (Fig. 2d,e). Tartrate-resistant acid phosphatase (TRAP) activity was markedly increased in the primary spongiosa of 2-month old *Gna13*^*f/f*^*LysM*-Cre mouse femur as compared with that of WT littermates (Fig. 2f–h). Histomorphometric parameters of distal femurs showed that, in the trabecular bone area, *Gna13*^*f/f*^*LysM*-Cre mice had ∼3-fold increased in osteoclast numbers per bone surface (N.Oc/BS) as compared with WT littermates (Fig. 2g). Interestingly, osteoclast number was even more drastically increased in the cortical bone area in the mutant mice (Fig. 2f,g). Similarly, X-ray and μ-CT analyses showed that skeletal mass was decreased in distal femurs of 2-month-old *Gna13*^*f/f*^*Ctsk*-Cre mice (Fig. 2i,j, Supplementary Fig. 2c). TRAP staining showed ∼6-fold increase in osteoclast numbers in femoral sections of *Gna13*^*f/f*^*Ctsk*-Cre mice as compared with that of WT controls (Fig. 2k, Supplementary Fig. 1d–f). Histomorphometry based on Trichrome staining showed that trabecular bone number was reduced without much change of osteoblast number and osteoblast surface per bone surface in the *Gna13*^*f/f*^*Ctsk*-Cre mice, confirming that the reduced bone density was not caused by decreased bone formation (Supplementary Fig. 2a,b). In fact, as measured by calcein labelling (Supplementary Fig. 2d), mineral apposition rate was increased by about 35% in *Gna13*^*f/f*^*Ctsk*-Cre mice. Consistently, serum alkaline phosphatase (ALP) was also increased by about 35% in *Gna13*^*f/f*^*Ctsk*-Cre mice (Supplementary Fig. 2e). The accelerated bone formation and remodelling might be caused by robust osteoclast formation. These results show that osteoclast-lineage-specific *Gna13* deletion promotes osteoclastic bone resorption leading to lower bone density *in vivo*.

### *Gna13*-depletion hyper-activates osteoclast

**Figure 3:**
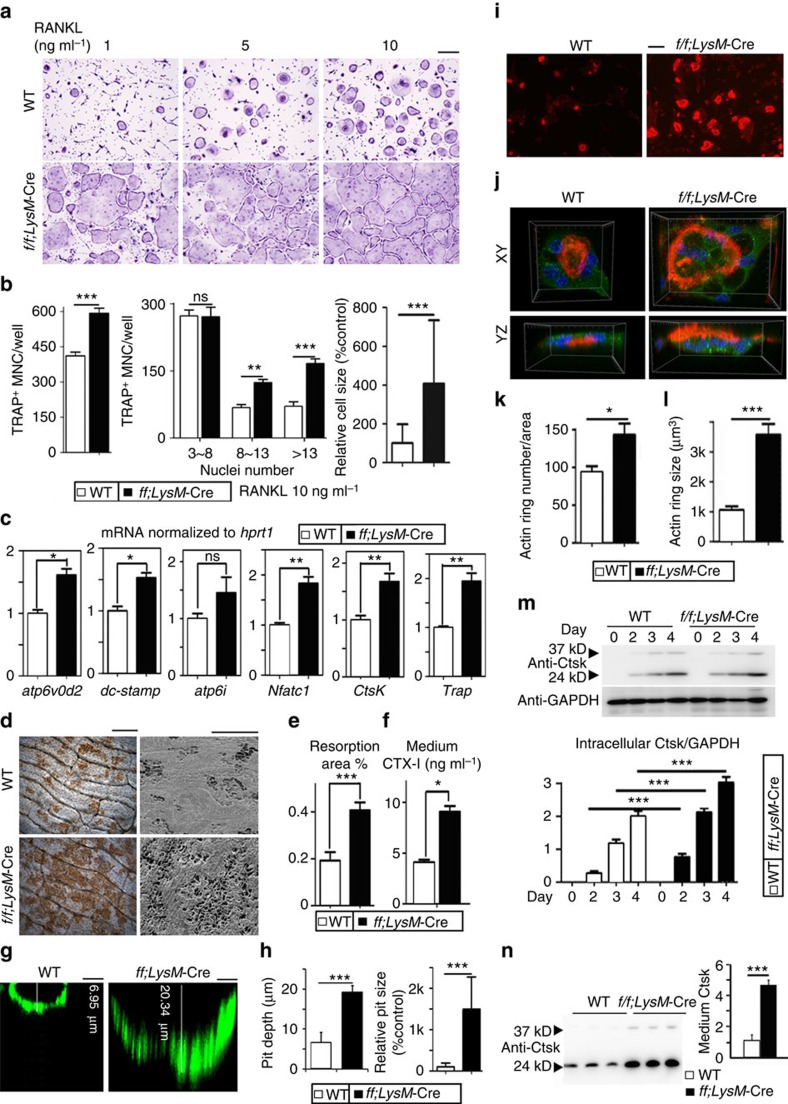
Loss of Gα13 promotes osteoclast formation and function. (**a**) TRAP staining to detect osteoclast formation from WT and *Gna13*^*f/f*^*LysM*-Cre (*f/f;LysM*-Cre) BMMs treated by M-CSF and different doses of RANKL for 5 days. (**b**) Quantification of cell number and relative cell size in **a**, RANKL=10 ng ml^−1^ group; *N*=6. TRAP^+^ MNC, TRAP positive multinucleated cell. (**d**) Quantitative reverse transcription–PCR analysis of *atp6v0d2*, *dc-stamp*, *atp6i*, *nfatc1*, *ctsk* and *trap* expression in WT and *Gna13*^*f/f*^*LysM*-Cre osteoclasts; *N*=6. (**e**) WGA (wheat germ agglutinin) staining and SEM (scanning electron microscope) analysis of bone slides to detect bone resorption pits. (**f**) Quantification of bone resorption area in **e**; *N*=6. (**g**) Confocal microscopy of FITC-WGA stained bone resorption pits. (**h**) Quantification of bone resorption pit depth and relative pit size in **g**. Culture medium CTX-I (type I collagen carboxy-terminal peptide) concentration measured by ELISA. (**i**) Fluorescence microscopy of F-actin ring stain in mature osteoclasts. (**j**) Confocal microscopy of F-actin ring (red)/Ctsk (green)/nuclei (blue) staining in mature osteoclasts. (**k**) Quantification of F-actin ring number in **i**; *N*=6. (**l**) Quantification of F-actin ring volume in **j**; *N*=30. (**m**) Western blot to detect intracellular Ctsk protein level, and its quantification (lower panel); *N*=3. (**n**) Western blot to detect Ctsk concentration in the culture medium, and its quantification (right panel); *N*=3. Results are expressed as mean±s.d.; *P≤0.05; **P≤0.01; ***P≤0.001 (Student's *t*-test or ANOVA analysis). Scale bars in **a**,**i**,**d**, 200 μm; Scale bar in **g**, 5 μm.

Given our data that *Gna13* deficiency *in vivo* led to an increase in osteoclast development (Fig. 2), we examined the effects of using lower doses of RANKL in osteoclastogenesis under *Gna13* deficiency. Unlike WT BMMs, *Gna13*-deficient cells formed osteoclasts when attended by permissive RANKL dose (one tenth of the optimum 10 ng ml^−1^ RANKL) (Fig. 3a) and formed 25% more osteoclasts at 10 ng ml^−1^ RANKL (Fig. 3a,b). Further, BMMs deficient in *Gna13* generated osteoclast earlier (∼day 3) than WT BMMs (∼day 5) (Supplementary Fig. 3a,b). *Gna13* deficiency not only increased the sensitivity of BMMs to RANKL, but also increased the size of osteoclasts (Fig. 3a,b). More osteoclasts with 3–8 nuclei or >13 nuclei were observed in the *Gna13*-deficient group (increased by 50% and 100%, respectively) (Fig. 3b). Consistently, gene expression analysis revealed that the expression of the osteoclast genes encoding *NFATc1, Ctsk, Acp5, Atp6v0d2* and *dc-stamp* was higher in *Gna13*^*f/f*^*LysM*-Cre, but not in WT, BMMs cultured with M-CSF and RANKL for 3 days (Fig. 3c). Importantly, among the aforementioned genes, *dc-stamp* and *Atp6v0d2* are known to have roles in osteoclast fusion^15^,^16^, indicating that Gα13 negatively regulates osteoclast formation by also promoting the fusion of mononucleated osteoclasts into multinucleated osteoclasts.

To further analyse the role of Gα13 in osteoclastic bone resorption, we performed *in vitro* bone resorption assays and analysed bone resorption pits by wheat germ agglutinin (WGA) staining and scanning electron microscope (Fig. 3d). Data showed that the total resorption area by *Gna13*^*f/f*^*LysM*-Cre osteoclasts was significantly higher than that of WT osteoclasts (Fig. 3e). Collagen I C-terminal telopeptide (CTX-I), released during bone resorption^17^, were drastically increased in *Gna13*^*f/f*^*LysM*-Cre cell culture medium (Fig. 3f). In addition, WGA-fluorescein isothiocyanate (FITC) staining showed that pits resorbed by *Gna13*^*f/f*^*LysM*-Cre osteoclasts are also larger (∼14-fold) and deeper (∼3-fold) than those by WT cells (Fig. 3g,h, Supplementary Fig. 4). Collectively, the results showed that Gα13 negatively regulated osteoclastic bone resorption.

Next, we co-stained the osteoclasts on bone slides with Rhodamine-conjugated-Phalloidin (red), to analyse F-actin ring formation, a critical structure of mature osteoclasts^18^, anti-Ctsk antibody (green) and Hoechst (blue) (Fig.3i,j). Data showed that *Gna13*-deficient osteoclasts exhibited 1.5-fold increased F-actin ring formation (Fig. 3i,k), and the average size of F-actin rings was also about threefold larger in these cells than control cells (Fig. 3j,l).

Furthermore, immunostaining showed a more polarized location of Ctsk in *Gna13*^*f/f*^*LysM*-Cre cells (Fig. 3j), indicating a change in Ctsk transportation and secretion. We thus analysed intracellular and medium Ctsk by western blot (Fig. 3m,n). Data showed a slight increase (∼1.5-fold) in intracellular Ctsk level in *Gna13*^*f/f*^*LysM*-Cre osteoclasts (Fig. 3m). However, secreted Ctsk concentration in the culture medium by *Gna13*^*f/f*^*LysM*-Cre osteoclasts was drastically elevated (∼4-fold) (Fig. 3n). These results demonstrate that *Gna13*-deficient osteoclasts had enlarged actin ring with increased Ctsk secretion, both of which would contribute to enhanced bone resorption besides enhanced osteoclast formation.

We performed TUNEL staining on day-6 osteoclasts (Supplementary Fig. 3c,e) and cytokine/serum-starved day-5 osteoclasts (Supplementary Fig. 3d,f), and similar apoptosis nuclei ratio are observed in the WT and mutant cells. Similar survival rate was observed in the WT and *Gna13*^*f/f*^*LysM*-Cre cells when the osteoclasts were extensively cultured for 7 days (Supplementary Fig. 3h,i). Data indicated that *Gna13*-deficiency might not influence osteoclast apoptosis. Further, acridine Orange staining showed that acidification is similar between WT and *Gna13*^*f/f*^*LysM*-Cre osteoclasts (Supplementary Fig. 3g).

### Gα13-RhoA brakes Akt-GSK3β-NFATc1 signalling

**Figure 4:**
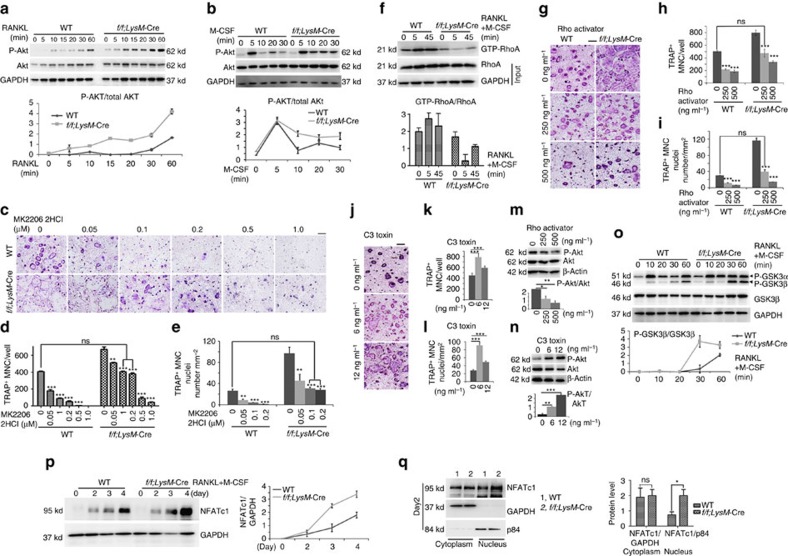
Knockout of *Gna13* enhances Akt-GSK3β-Nfatc1 signalling through RhoA. (**a**) Western blot analysis to detect Akt phosphorylation induced by RANKL in WT and *Gna13*^*f/f*^*LysM*-Cre (*f/f;LysM*-Cre) monocytes/macrophages. *N*=3. (**b**) Western blot analysis to detect Akt phosphorylation induced by M-CSF in WT and *Gna13*^*f/f*^*LysM*-Cre monocytes/macrophages. Lower panel in **a**–**b**, Quantification of phospho-Akt level versus total Akt level; *N*=3. (**c**) TRAP staining to detect osteoclastogenesis of WT and Gna13-deficient osteoclasts treated by MK2206 2HCl at different doses during differentiation. (**d**) Quantification of TRAP^+^ MNC number per well in **c**; *N*=6. (**e**) Quantification of TRAP^+^ nuclei number per mm^2^ in **c**; *N*=6. (**f**) RhoA activity in WT and *Gna13*^*f/f*^*LysM*-Cre pre-osteoclasts upon RANKL and M-CSF stimulation, and its quantification; *N*=3. (**g**) TRAP staining of WT and *Gna13*^*f/f*^*LysM*-Cre osteoclasts treated by Rho activator II at different doses during differentiation. (**h**) Quantification of TRAP^+^ MNC number per well in **g**; *N*=6. (**i**) Quantification of TRAP^+^ nuclei number per mm^2^ in **g**; *N*=6. (**j**) TRAP staining of WT and *Gna13*^*f/f*^*LysM*-Cre osteoclasts treated by different doses of RhoA inhibitor (C3 toxin) (0, 6, 12 ng ml^−1^) during differentiation. (**k**) Quantification of TRAP^+^ MNC number per well in **j**; *N*=6. (**l**) Quantification of TRAP^+^ nuclei number per mm^2^ in **j**; *N*=6. (**m**) Akt phosphorylation in pre-osteoclasts treated different doses of Rho activator II. (**n**) Akt phosphorylation in pre-osteoclasts treated different doses of C3 toxin (0, 1, 3, 6 ng ml^−1^). Lower panel in **m**–**n**, Quantification of phospho-Akt level versus total Akt level; *N*=3. (**o**) GSK3β phosphorylation induced by RANKL and M-CSF. Lower panel, Quantification of phospho-GSK3β level versus total GSK3β level; *N*=3. (**p**) Western blot analysis of NFATc1 expression in WT and *Gna13*^*f/f*^*LysM*-Cre osteoclast precursors. (**q**) NFATc1, GAPDH (cytoplasm loading control) and p84 (nuclei loading control) in Nuclei and cytoplasm lysates of WT and *Gna13*^*f/f*^*LysM*-Cre pre-osteoclasts. Right panels in **p**–**q**, Quantification of NFATc1 level versus loading control; *N*=3. Results were expressed as mean±s.d.; *P≤0.05; **P≤0.01; ***P≤0.001 (Student's *t*-test or ANOVA analysis). Scale bars, 200 μm.

RANKL and M-CSF orchestrate multiple signalling pathways to promote osteoclast formation^1^,^2^,^3^. Expression of RANKL receptor (RANK) and M-CSF receptor (cFms) were similar in WT and *Gna13*-deficient cells (Supplementary Fig. 3j,k). To analyse signal transduction in response to RANKL or M-CSF stimulation (including Akt, p38, JNK and Erk activation), BMMs were starved for 5 h and then stimulated with RANKL or M-CSF for 5–60 min (Fig. 4a,b). Akt phosphorylation was enhanced in *Gna13*^*f/f*^*LysM*-Cre cells by either RANKL or M-CSF as compared with WT BMMs (Fig. 4a,b). Nonetheless, activation of the p38, JNK and Erk signalling pathways were similar between WT and *Gna13*^*f/f*^*LysM*-Cre cells (Supplementary Fig. 5).

Given the drastic increase in Akt activation, we then applied the Akt inhibitor MK2206 2HCl to attenuate Akt activity, which inhibited osteoclastogenesis in a dose-dependent manner (Fig. 4c–e). Notably, *Gna13*-deficient cells were more resistant to the Akt inhibitory effect on osteoclastogenesis. In the presence of 0.1∼0.2 μM MK2206 2HCl (Fig. 4d,e), *Gna13*-deficient cells formed osteoclasts at normal level comparatively to WT cells. The results indicate that *Gna13* deficiency promotes osteoclastogenesis by increasing Akt activity.

Studies have demonstrated that Gα13 activates RhoA through RhoGEF (p115RhoGEF, LARG *et al*.)^11^,^12^, and RhoA can inhibit Akt activity^19^. Consistently, RhoA activity was dramatically downregulated in *Gna13*-deficient cells (Fig. 4f). In all, 250 ng ml^−1^ Rho activator II corrected the hyper-activation of *Gna13*-deficient osteoclasts (Fig. 4g–i). In addition, Rho activator II could attenuate Akt activation in osteoclasts (Fig. 4m). On the other hand, in the presence of 6–12 ng ml^−1^ Rho inhibitor, cell-permeable C3 toxin, osteoclast differentiation was enhanced (Fig. 4j–l) and Akt activation was also increased (Fig. 4n). Thus, Gα13 regulated Akt activity through RhoA so as to promote osteoclastogenesis.

It has been established that the PI3K/Akt-GSK3β-NFATc1 signalling cascade is critical for osteoclast differentiation^20^,^21^. Specifically, Akt can inhibit GSK3β, which can promote NFATc1 translocation from the nucleus into the cytoplasm and thereby abrogate osteoclast differentiation. Thus, we characterized GSK3β phosphorylation level and NFATc1 nuclei translocation in WT and *Gna13*^*f/f*^*LysM*-Cre cells. We found that GSK3α/β phosphorylation was hyper-activated in *Gna13*^*f/f*^*LysM*-Cre monocyte/macrophage in response to RANKL and M-CSF (Fig. 4o). NFATc1 expression increased during osteoclastogenesis in WT and *Gna13*^*f/f*^*LysM*-Cre cells, and more quickly in *Gna13*^*f/f*^*LysM*-Cre cells (Fig. 4p). Consistently, while similar amounts of NFATc1 were observed in the cytoplasm, much more NFATc1 was observed in the *Gna13*^*f/f*^*LysM*-Cre nucleus than WT (Fig. 4q). The results demonstrated that Gα13 mediates Akt-GSK3β-NFATc1 signalling to promote osteoclast gene expression.

### Gα13CA inhibits osteoclastogenesis

**Figure 5:**
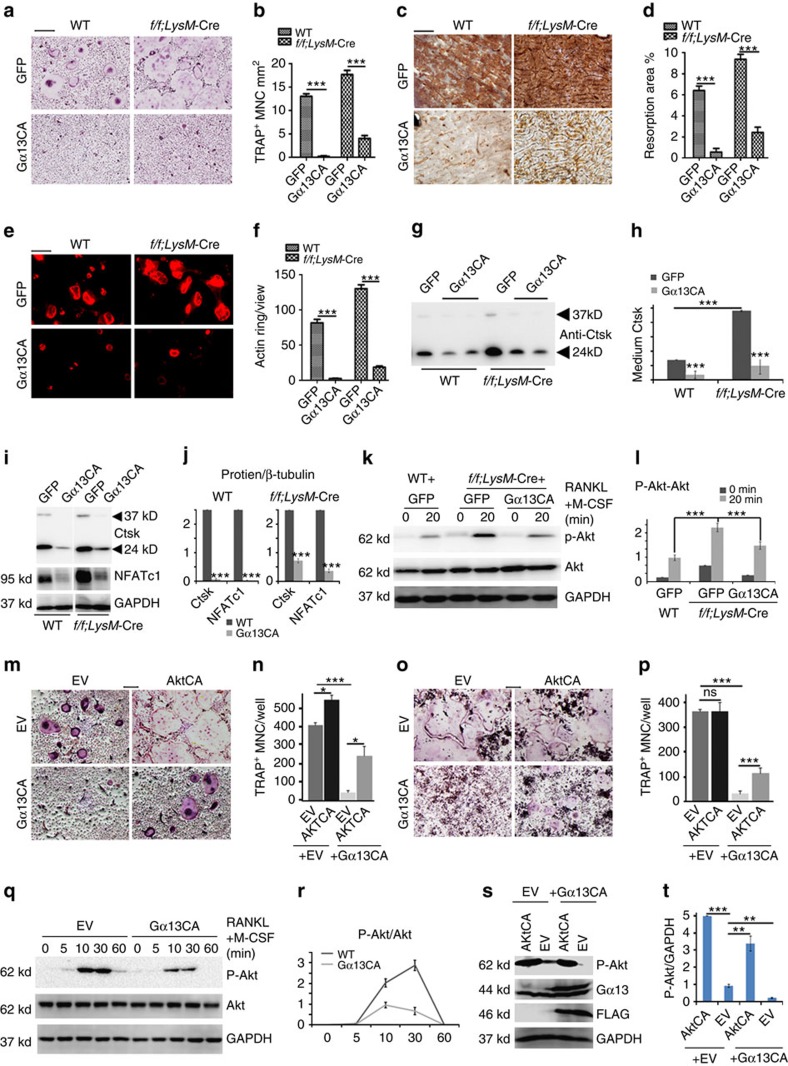
Gα13 gain-of-function inhibits osteoclast formation and function *in vitro*. (**a**) TRAP staining to detect osteoclastogenesis of WT and *Gna13*^*f/f*^*LysM*-Cre (*f/f;LysM*-Cre) cells transfected with lentivirus overexpressing green fluorescent protein (GFP) and Gα13CA. (**b**) Quantification of TRAP^+^ MNC number per mm^2^ in **a**; *N*=6. (**c**) WGA staining to detect bone resorption of WT and *Gna13*^*f/f*^*LysM*-Cre cells overexpressing GFP and Gα13CA. (**d**) Quantification of bone resorption area per total area in **c**; *N*=6. (**e**) Rhodamine-conjugated-Phalloidin staining to detect F-actin ring formation of WT and *Gna13*^*f/f*^*LysM*-Cre cells transfected with lentivirus overexpressing GFP and Gα13CA. (**f**) Quantification of F-actin ring number per view in **e**; *N*=6. (**g**,**i**,**k**) Western blot to detect medium Ctsk (**g**), osteoclast-specific gene expression (**i**) Akt phosphorylation induced by RANKL and M-CSF (**k**) in WT and *Gna13*^*f/f*^*LysM*-Cre osteoclasts (overexpressing GFP or Gα13CA). (**h**,**j**,**l**) Quantification of protein level in **g**–**l**; *N*=3. (**m**,**o**) TRAP staining to detect osteoclastogenesis of BMMs (**m**) and RAW264.7 cells (**o**), which were transfected with control retrovirus and retrovirus overexpressing 3xFLAG-Gα13CA, AktCA or 3xFLAG-Gα13CA+AktCA. (**n**,**p**) Quantification of TRAP^+^ MNC per well in **n**,**m** and in **p**,**o**; *N*=6. (**q**,**r**) Western blot analysis of RANKL induced Akt phosphorylation in RAW264.7 cells transfected with control retrovirus and retrovirus overexpressing Gα13CA, and its quantification; *N*=3. (**s**,**t**) Western blot confirmation of AktCA and Gα13CA overexpression in RAW264.7 cells, and its quantification; *N*=3. EV, empty vesicle control. Results were expressed as mean±s.d.; *P≤0.05; **P≤0.01; ***P≤0.001 (Student's *t*-test or ANOVA analysis). Scale bars, 200 μm.

To confirm the inhibitory effect of Gα13 on osteoclastogenesis, we overexpressed a constitutively active form of Gα13 (Gα13CA) in BMMs and RAW264.7 cells (Fig. 5). Moreover, we overexpressed Gα13CA in WT and *Gna13*^*f/f*^*LysM*-Cre pre-osteoclasts using lentivirus^22^. A successful infection (>90% infection) was assessed by green fluorescent protein expression in control groups (Supplementary Fig. 6a,c), and Gα13CA overexpression was confirmed by western blot (Supplementary Fig. 6b,d,e). Importantly, Gα13CA overexpression drastically blocked osteoclast fusion, inhibited bone resorption and impaired F-actin ring formation in WT and *Gna13f/f;LysM*-Cre cells (Fig. 5a–f). Additionally, the secretion of Ctsk was decreased after Gα13CA overexpression in WT and *Gna13*-deficient osteoclasts (Fig. 5g,h). Ctsk and NFATc1 protein levels were also decreased after Gα13CA overexpression (Fig. 5i,j). Furthermore, Gα13CA overexpression attenuated Akt activation in *Gna13*^*f/f*^*LysM*-Cre cells, but did not affect the activation of p38 and ERK (Fig. 5k,l; Supplementary Fig. 7a). Gα13CA overexpression in WT BMMs also drastically inhibited osteoclastogenesis (Fig. 5m left panels; 5n). Interestingly, overexpression of a constitutively active form of Akt (AktCA) could partially rescue the inhibitory effects of Gα13CA (Fig. 5m right panels; 5n). These results demonstrate that Gα13CA inhibited osteoclastogenesis by inhibiting Akt activation in BMMs.

Furthermore, we infected RAW264.7 cells with the same retrovirus we used in BMMs in Fig. 5m,o. Similarly, Gα13CA overexpression inhibited osteoclast-like cell formation (Fig. 5o left panels; 5p), which could be rescued by AktCA overexpression (Fig. 5o right panels; 5p). Akt activation was greatly decreased in RAW264.7 cells overexpressing Gα13CA (Fig. 5q,r), while the activation of p38 and Erk remains unchanged (Supplementary Fig. 7b). The overexpression of Gα13CA and AktCA was confirmed by western blot (Fig. 5s,t). The results indicated that Gα13CA also inhibited osteoclast-like cells formation from the RAW264.7 cells by inhibiting Akt phosphorylation.

### Gα13CA protects mice from pathological bone loss

**Figure 6:**
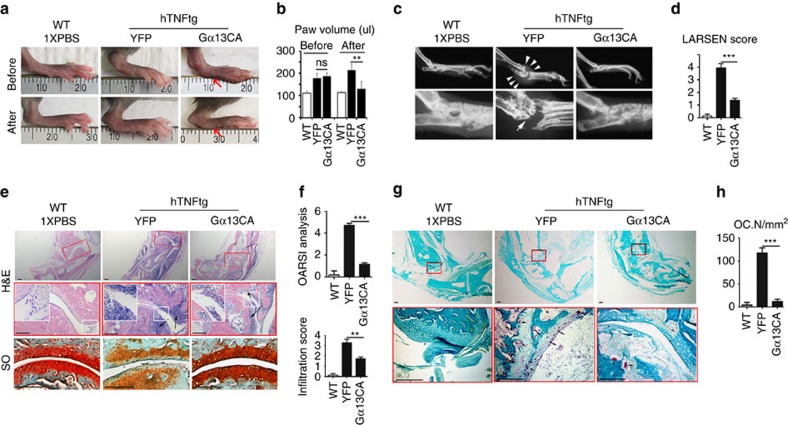
Gα13 protects Rhematoid arthritis mice from inflammatory bone loss. (**a**–**h**) Histology analysis of 18-week-old male WT and hTNFtg Rheumatoid arthritis mouse ankles. hTNFtg mouse ankles were injected with AAV-Gα13CA or AAV-YFP. (**a**) Photographic images before and after AAV treatment. The red arrows showed paw swelling is relieved after AAV-Gα13CA treatment. (**b**) Quantification of hind paw volume in **a**; *N*=8. (**c**,**d**) Radiographic images; White arrows mark bone destruction. (**d**) Quantification of bone destruction (Larsen grade) in **a**; *N*=8. (**e**) H&E staining (black arrows mark monocyte infiltration) and Safranin O (SO) staining (black arrows mark articular cartilage damage). (**f**) Quantification of cartilage damage (OARSI grade) and inflammation (infiltration score) in **e**; *N*=8. (**g**) TRAP staining; black arrows mark TRAP positive cells. (**h**) Quantification of osteoclast number (Oc.N) in **g**; *N*=8. Results were expressed as mean±s.d.; *P≤0.05; **P≤0.01; ***P≤0.001 (Student's *t*-test). scale bars in **e**,**g** 200 μm.

**Figure 7:**
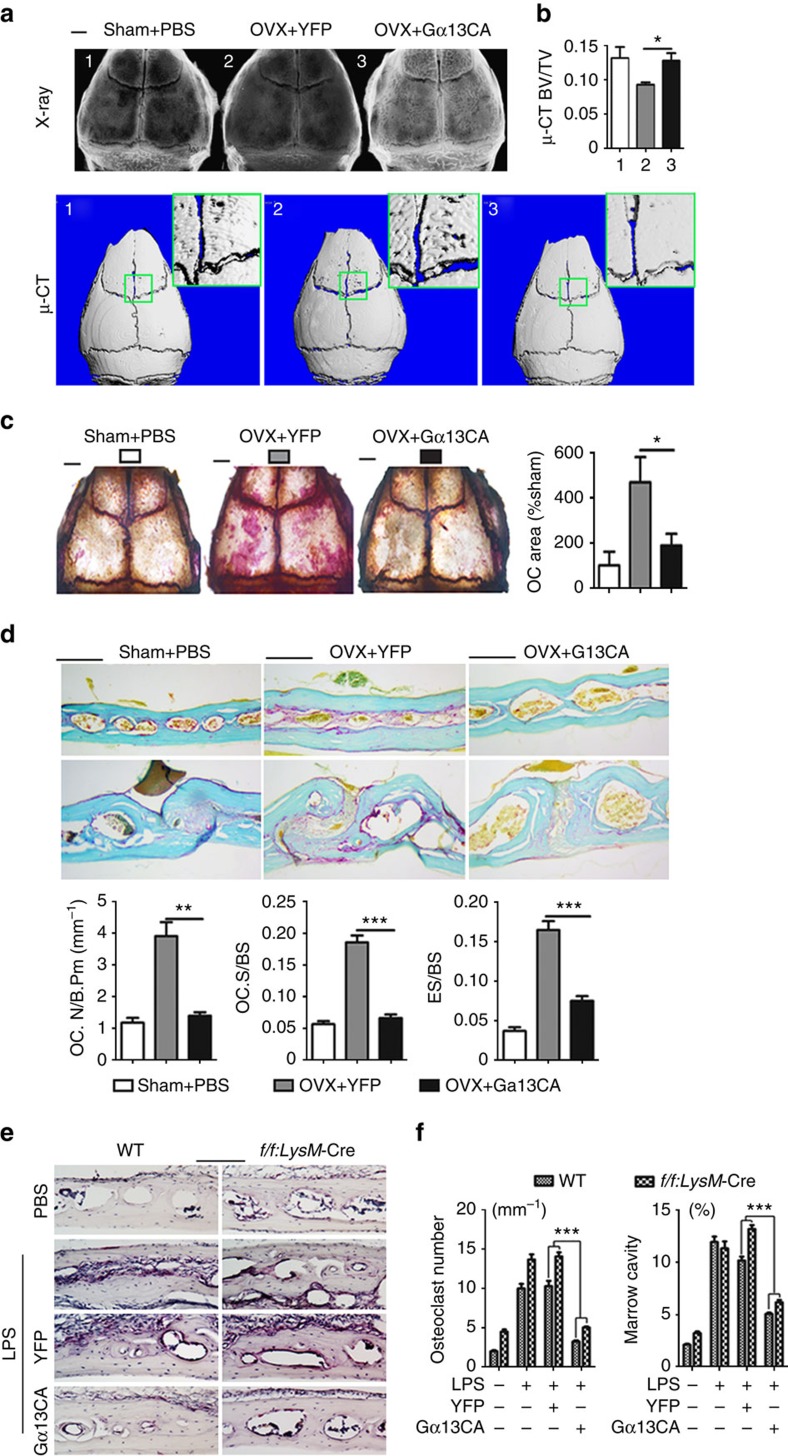
Gα13 protects mice from OVX- and LPS-induced bone loss. (**a**–**d**) Histology analysis of calvarial bones from 13-week-old female sham-operated mice (control), and ovariectomized (OVX) mice injected with AAV-YFP or AAV-Gα13CA. (**a**) Radiographic (higher panels) and μ-CT analysis (lower panels). High-magnification images are co-presented in the right corners. (**b**) Quantification of bone volume per tissue volume (BV/TV); *N*=3. (**c**) Whole calvaria TRAP staining; Quantification of relative TRAP positive area is co-presented on the right panel; *N*=4. (**d**) TRAP staining of calvaria frozen sections. Quantification of osteoclast and bone resorption metrics are presented in lower panel; OC.N/B.Pm, osteoclast number per bone perimeter; OC.N/BS, osteoclast number per bone surface; ES/BS, eroded surface per bone surface; *N*=4. (**e**) TRAP staining using frozen sections of calvarial bones from 8-week-old male WT and *Gna13*^*f/f*^*LysM*-Cre (*f/f;LysM*-Cre) mice treated with PBS, LPS, LPS+AAV-YFP or LPS+AAV-Gα13CA. (**f**) Quantification of TRAP-positive cells per bone surface and marrow cavity area in **e**; *N*=6. Results were expressed as mean±s.d.; *P≤0.05; **P≤0.01; ***P≤0.001 (Student's *t*-test or ANOVA analysis). scale bars in **a**,**c** 200 μm; scale bars in **d**,**e**, 1 mm.

To investigate Gα13 clinical implication potential, we overexpressed Gα13CA locally in mice using adeno-associated virus (AAV) gene expression to investigate its effects in protecting against bone degradation. AAV vectors have advantages compared with many other viral and non-viral based gene delivery platforms in both function and safety for correcting genetic-based diseases^23^. To test the use of AAV-Gα13CA, we utilized three disease models of bone loss: the human tumour-necrosis factorα (hTNFα)-transgene expressing mouse model of autoimmune arthritis^24^ (named TNFα-RA) (Fig. 6), ovariectomized (OVX) animal model of osteoporosis, (Fig. 7a–d) and calvaria-adjacent lipopolysaccharide (LPS)-injection mouse model of osteolysis (Fig. 7e,f)^25^. Successful infection of AAV *in vivo* was monitored by yellow fluorescent protein (YFP) expression (Supplementary Figs 8 and 9a). Successful overexpression of G13CA *in vivo* was confirmed by immunofluorescence staining (Supplementary Fig. 9b).

TNFα-RA mice were accompanied by excessive osteoclast formation and bone destruction (Fig. 6). Local administration of AAV-Gα13CA to ankles greatly reduced bone loss, as assessed by X-ray in the TNFα-RA mice as compared with those injected with AAV-YFP (Fig. 6c,d). Not surprisingly, osteoclast number was dramatically decreased in TNFα-RA mice with local injection of AAV-Gα13CA to ankles (Fig. 6g,h). Unexpectedly, local administration of AAV-Gα13CA also relieved ankle swelling (Fig. 6a,b), cartilage destruction (Fig. 6e lower panels, Fig. 6f) and inflammation cell infiltration (Fig. 6e upper and middle panels, Fig. 6f) in TNFα-RA mice. Hence, reduced bone loss and decreased osteoclast number might be a combined effect of Gα13CA and inhibited inflammation.

OVX-induced bone loss and osteoclast number increased due to estrogen depletion (Fig. 7a–d, sham+PBS group compared with OVX+YFP group). OVX mice were subjected to calvaria-adjacent subcutaneous injection of AAV-YFP or AAV-Gα13CA. As assessed by X-ray and u-CT, application of AAV-Gα13CA reduced OVX-induced bone loss (Fig. 7a,b). As assessed by TRAP staining of whole calvarial and calvarial sections, AAV-Gα13CA dramatically reduced OVX-induced osteoclast number increase (Fig. 7c,d) as compared with YFP group.

In the LPS-injection model, both WT and *Gna13*^*f/f*^*LysM*-Cre mice were subjected to calvaria-adjacent subcutaneous injection of LPS or PBS control (Fig. 7e,f). Compared with local injection of PBS, LPS induced a dramatic increase in osteoclast number and bone resorption (Fig. 7e,f). The absence of Gα13 further promoted the induction of osteoclastogenesis and bone loss in both PBS and LPS treated mice (Fig. 7e,f). Local administration of AAV-Gα13CA to calvaria had a marked therapeutic effect on osteoclast formation and bone destruction by LPS, compared with local injection of AAV-YFP control (Fig. 7e,f). These results confirm that it is possible to overexpress Gα13CA *in vivo* to target pathologic bone loss.

Full images of western blots are presented in Supplementary Fig. 10.

## Discussion

**Figure 8:**
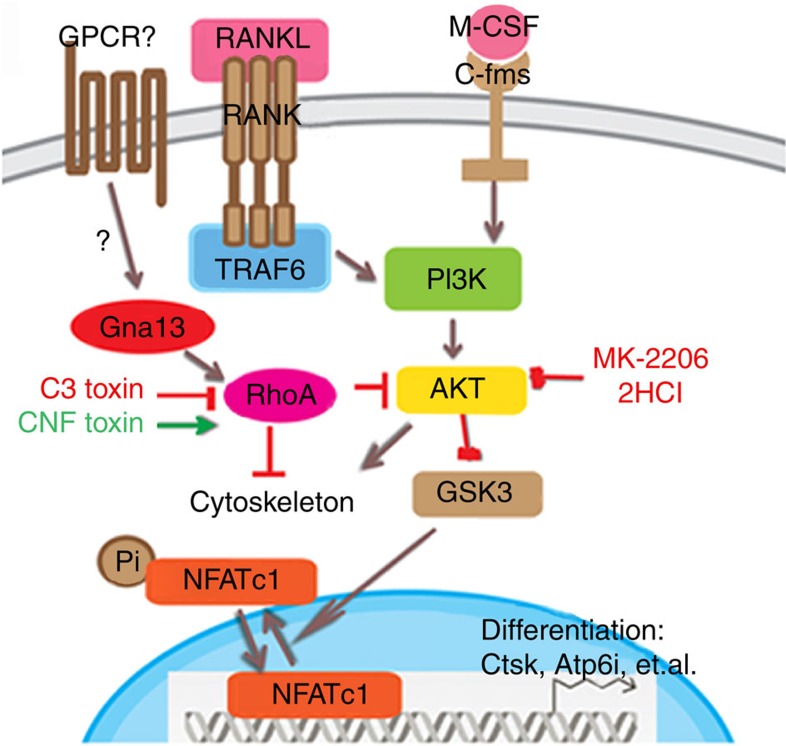
Working model of Gα13 as a negative regulator in osteoclast. CNF toxin, Rho activator; C3 toxin, Rho inhibitor.

We proposed a model that Gα13-RhoA antagonizes osteoclast formation and activity by attenuating the Akt-GSK3β-NFATc1 signalling axis (Fig. 8). Briefly, as a downstream of RANK and c-Fms, Akt phosphorylates and inactivates GSK3β, which has a role in NFATc1 nuclei exportation, so as to promote osteoclast differentiation; Meanwhile, Gα13 activates RhoA which in turn inhibits Akt phosphorylation and activity, so as to antagonize the over-activation of osteoclast (Fig. 8). The critical role of PI3K-Akt activity in osteoclast differentiation and activation is well-documented^20^,^21^,^26^,^27^,^28^,^29^. Although several endogenous factors that oppose osteoclast differentiation were described, most are downregulated under osteoclastogenic condition. In contrast, Gα13 is induced by the combined stimulation of RANKL and M-CSF, and in turn inhibit osteoclast differentiation so as to avoid over-activated osteoclast and excessive bone loss. Hence, our data reveal that Gα13 is an intrinsic control mechanism during osteoclastogenesis. In addition, we comprehensively study and report a signalling cascade (Gα13-RhoA-Akt-GSK3β-NFATc1) that negatively regulates osteoclastogenesis and osteoclast function. Most importantly, our results from both loss-of-function and gain-of-function strategies proved that Gα13 is a key negative regulator and main switch in osteoclastogenesis.

It was reported that RhoA activity is essential for podosome formation and osteoclastic bone resorption^30^. On the other hand, it was also reported that increased RhoA activity destabilizes the sealing zone in osteoclasts, and inhibition of RhoA stabilizes the sealing zone correlating with acetylation and stabilization of the microtubule network in these cells^31^,^32^,^33^,^34^. Our study showed that inhibition of RhoA activity through Gα13 knockout, RhoA inhibitor and RNA interference favours osteoclastogenesis, while RhoA activator inhibits osteoclastogenesis. Hence, these studies suggest that RhoA is the downstream effector of Gα13 to negatively regulate osteoclastogenesis. Our finding is underscored by genetic evidences that have suggested an important role for the RhoGEF-RhoGTPase (for example, Arhgef3-RhoA) pathway in osteoporosis^35^,^36^,^37^,^38^. Genome-wide linkage studies have identified chromosome region 3p14-p21, in which *RHOA* gene was located, as a quantitative trait locus for BMD^35^,^36^. One *RHOA* single-nucleotide polymorphism (SNP) rs17595772, and one *ARHGEF3* SNP were reported to be significantly associated with decreased BMD) in postmenopausal women^37^,^38^. However, the mechanisms underlying the correlation between RhoGEF-RhoGTPase pathway and BMD remain to be explored.

Current anti-resorptive agents (for example, bisphosphonates and denosumab) are effective but far from ideal. The major problem of bisphosphonates is their permanent deleterious affect on normal bone remodelling. Agents preventing bone resorption by inhibiting late differentiation of osteoclasts without affecting normal bone remodelling are more desired, which are unlikely to interfere with the coupling of osteoblastic bone formation to osteoclastic bone resorption, critical for maintaining normal bone homeostasis^39^. Here we showed that overexpression of Gα13CA markely inhibits osteoclast formation and bone resorption both *in vitro* and *in vivo*. Importantly, although multi-nuclear cell formation was blocked, Gα13CA over-expressing monocytes were TRAP^+^ stained (Fig. 5a), indicating that Gα13CA regulated osteoclast differentiation at a late stage and could be a good target for bone loss without affecting normal bone remodelling. Interestingly, Gα13 might have dual functions in inflammatory disease models (TNFα-RA and LPS-injection models), by concurrently inhibiting osteoclast differentiation and inflammation so as to achieve the ultimate outcome of bone loss protection. Hence, activating Gα13, an endogenous osteoclast inhibitor, may serve as a novel therapeutic approach targeting bone loss in various bone pathological states.

## Methods

### Mice

*Gna13*^flox/flox^ (*Gna13*^*f/f*^) mice were provided by Dr S. Offermanns (Max-Planck-Institute for Heart and Lung Research, Bad Nauheim, Germany)^14^. *LysM*-Cre transgenic mice were purchased from the Jackson laboratory. *Ctsk*-Cre knock-in mice were provided by Dr S. Kato (University of Tokyo, Tokyo, Japan)^40^. To obtain mice with *Gna13*-deficient macrophages, *Gna13*^*f/+*^*LysM*-Cre males were bred with *Gna13*^*f/+*^*LysM*-Cre females. To obtain mice with Gna13-deficient osteoclasts, *Gna13*^*f/+*^*Ctsk*-Cre males were bred with *Gna13*^*f/f*^ females. In all experiments, *Gna13*^*f/+*^*LysM*-Cre or *Gna13*^*f/+*^*Ctsk*-Cre mice were compared with their WT littermates (*Gna13*^*f/+*^, *Gna13*^*f/f*^, *Gna13*^*+/+*^*LysM*-Cre or *Ctsk*-Cre mice). All mice were on a pure C57BL/6 background. All animal studies were approved by the Institutional Animal Care and Use Committee (IACUC) at University of Alabama at Birmingham (UAB), Birmingham, USA.

### GeneChip analysis

Human peripheral blood mononuclear cells were cultured for 7 days with human RANKL and human M-CSF. GeneChip data were analysed using Affymetrics scanner and accompanying gene expression software.

### Radiographic analysis.

For X-ray analysis, excised 2-month-old mouse femurs were scanned using a high-resolution soft X-ray system (Faxitron Model MX-20) at 30 kV. For Micro-Computed Tomography (μ-CT) analysis, excised 2-month-old mouse femurs were scanned using the Scanco CT40 desktop cone-beam μ-CT scanner (Scanco Medical AG, Brüttisellen, Switzerland). The scan of the trabecular bone was performed from the growth plate, and consisted of 310 slices (12 μm per slice) and 200 slices were analysed using the CT Evaluation Program (v5.0A, Scanco Medical). The scan and analysis of the cortical bone was performed at the midshaft of the femur and consisted of 25 slices (12 μm per slice).

### Histology

Histology was performed as previously described^40^,^41^. Briefly, the femurs of 2-month-old mice were fixed with 70% ethanol followed by plastic embedding and Golden trichrome staining, or with 10% neutral buffered formalin followed by decalcification in 10% EDTA for 2 weeks, paraffin embedding and TRAP staining using Phosphatase, Leukocyte Acid (TRAP) kit (Sigma-Aldrich, 387A-1KT). Osteoclastic and osteoblastic perimeters were measured and analysed using Osteomeasure in a blinded fashion.

### *In-vivo* calcein labelling

Calcein labelling was previously described^41^. Briefly, 2-month-old mice were intraperitoneally injected with 20 mg kg^−1^ of calcein in a 2% sodium bicarbonate solution, 8 days and 2 days before killing of mice. Calvarias were fixed in 4% PFA, soaked in 30% glucose in PB, embedded in OCT and frozen sectioned. Mineral apposition rate is the distance between the midpoints of the two labels divided by the time between the midpoints of the interval.

### Serum ALP assay

Two-month old mouse serum was collected after 6-hour fasting, and the serum ALP activity was detected and quantified using Alkaline Phosphatase Assay Kit (Colorimetric) (ab83369) according to the manufacturer's instructions.

### *In vitro* osteoclast differentiation assay

Mature osteoclasts were generated as described^10^. Briefly, isolated BMMs from C57BL/6 mice were cultured in MEM α (pH 6.9) containing 10% FBS, 10 ng ml^−1^ recombinant RANKL and 10 ng ml^−1^ recombinant M-CSF for 5 days. Recombinant murine M-CSF and RANKL were obtained from R&D systems, Inc. Cell culture medium was obtained from Gibco, Life Technologies Corporation. Mature osteoclasts were characterized by staining for TRAP activity using a commercial kit (Sigma-Aldrich, 387A-1KT) and TRAP^+^ MNCs were enumerated per well in a 24-well plate.

### *In vitro* bone resorption assay

For bone resorption assay, osteoclasts were seeded on bovine bone slides. Concentration of bovine cross-linked C-telopeptide of type I collagen (CTX-1) in the medium was measured using CrossLaps for Culture ELISA (CTX-I) kit (Immunodiagnostic Systems Limited) following the manufacturer's instructions. Bone resorption pits were sonicated in PBS, stained with 2 ug ml^−1^ WGA-lectin (Sigma-Aldrich, L-3892) for two hours and then using DAB peroxidase (horseradish peroxidase) Substrate Kit (Vector laboratories, SK-4100) (ref. 42). To analyse bone resorption pits depth and volume, bone slides were sonicated in PBS, stained with 2 ug ml^−1^ FITC-WGA (Sigma-Aldrich, L-4895) staining for 2 h and visualized by confocal microscopy^43^.

### Cellular immunofluorescent staining

For F-actin ring staining, osteoclasts on bovine bone slides were incubated with 2 U ml^−1^ Texas Red-X Phalloidin (Life Technologies, T7471). For anti-Ctsk immunofluorescence stain, osteoclasts on bovine bone slides were fixed by 4% formaldehyde, permeabilized with 0.2% Triton X-100, blocked with 5% goat serum and 100 μg ml^−1^ unconjugated AffiniPure Fab Fragment Goat Anti-Mouse IgG (H+L) (Jackson ImmunoResearch Labs), incubated with mouse anti-mouse Ctsk primary antibody (1:100, Santa Cruz sc-48353) and then with FITC goat anti-mouse IgG (H+L) secondary antibody. Nuclei were visualized with 1 μg ml^−1^ DAPI (4',6-diamidino-2-phenylindole; Sigma-Aldrich, D9542). Three-dimensional images were taken by confocal microscopy and constructed by Imaris software^44^.

### Inhibitors and activators treatment.

MK-2206 2HCl was purchased from Selleckchem Inc. (Cat# S1078). Rho Inhibitor I (cell-permeable exoenzyme C3 transferase, or cell-permeable C3 toxin) was purchased from Cytoskeleton Inc. (Cat# CT04). Rho activator II was purchased from Cytoskeleton Inc. (Cat# CN03). Stock solutions were prepared according to the manufacturer's instructions. During differentiation from BMMs to mature osteoclasts, the cells were treated continuously by those inhibitors or activators at indicated doses.

### Quantitative reverse transcription–PCR (qPCR) assay

Total RNA was isolated from cultured cells with TRIzol reagent (Life Technologies, 15596018). Mouse complementary DNA (cDNA) was reverse-transcribed from 0.5 μg total RNA with SuperScript VILO Master Mix (Life Technologies, 11755050). Quantitative reverse transcription–PCR performed on StepOne Real-Time PCR System (Life Technologies, Applied Biosystems) using the TaqMan Gene Expression assays (Life Technologies, Applied Biosystems) or SYBR Green reagents (Life Technologies, 4385610). Taqman probe numbers and Primer sequences are as followings: *Ctsk* (Mm00484039_m1); *Acp5* (Mm00475698_m1), *Atp6i* (Mm00469394_m1), *Nfatc1* (Mm00479445_m1), *Hprt1* (Mm00446968_m1), *Hprt1* (5′-GGTGGAGATGATCTCTCAACTTTAA-3′, 5′-AGGAAAGCAAAGTCTGCATTGTT-3′), *Atp6v0d2* (5′-GAAGCTGTCAACATTGCAGA-3′, 5′-TCACCGTGATCCTTGCAGAAT-3′), *DC-stamp* (5′-GGGGACTTATGTGTTTCCACG-3′, 5′-ACAAAGCAACAGACTCCCAAAT-3′), *Gna13* (5′-TGCTGGTAGATGCCCGAGA-3′, 5′-GATCGTAGGCATTCTGTATACCA-3′), *β-actin* (5′-TGACGTTGACATCCGTAAAGAC-3′, 5′-TGCTAGGAGCCAGAGCAGTAA-3′).

### Western blotting and phosphorylation assay

Cell lysates preparation and SDS–polyacrylamide gel electrophoresis /western blotting analysis were carried out according to a standard protocol^10^,^45^. For the phosphorylation assay, BMMs and pre-osteoclasts (derived from BMMs with 2-day treatment of RANKL and M-CSF) were starved for 5 h and stimulated with RANKL or M-CSF for different times. Proteins were harvested in cell lysis buffer supplemented with proteinase inhibitor cocktail (Sigma-Aldrich Co. LLC, 1:100) and phosphatase inhibitor cocktail 2 (P-5726, Sigma-Aldrich Co. LLC, 1:100). To extract nuclear and cytoplasmic proteins, NE-PER Nuclear and Cytoplasmic Extraction Reagent Kit (Thermo Fisher Scientific Inc) was used. Western blot was performed using following antibodies:, anti-p84 mouse IgG (GeneTex GTX70220, 1:1,000), anti-IκBα rabbit IgG (Cell Signaling Technology 4812, 1:1,000), anti-phospho-IκBα (Ser32) rabbit IgG (Cell Signaling Technology 2859, 1:1,000), anti-p38 rabbit IgG (Cell Signaling Technology 9212, 1:1,000), anti-phospho-p38 (Thr180/Tyr182) rabbit IgG (Cell Signaling Technology 4631, 1:1,000), anti-Erk rabbit IgG (Cell Signaling Technology 4370, 1:1,000), anti-phospho-Erk (Thr202/Tyr204) rabbit IgG (Cell Signaling Technology 4695, 1:1,000), anti-Akt rabbit IgG (Cell Signaling Technology 4685, 1:1,000), anti-phospho-Akt (Thr308) rabbit IgG (Cell Signaling Technology 2965, 1:1,000), anti-JNK rabbit IgG (Cell Signaling Technology 9251, 1:1,000), anti-phospho-JNK (Thr183/Tyr185) rabbit IgG (Cell Signaling Technology 9258, 1:1,000), anti-c-Jun rabbit IgG (Cell Signaling Technology 9165), anti-phospho-c-Jun (Ser73) rabbit IgG (Cell Signaling Technology 9164, 1:1,000), anti- GSK3β rabbit IgG (Cell Signaling Technology 9315, 1:1,000), anti-phospho-GSK3α/β (Ser21/9) rabbit IgG (Cell Signaling Technology 9331, 1:1,000), anti-Gα13 rabbit IgG(Santa Cruz sc-410, 1:500), anti-Ctsk mouse IgG (Santa Cruz sc-48353, 1:500), anti-NFATc1 mouse IgG (Santa Cruz sc-7294, 1:500), anti-GAPDH mouse IgG (Santa Cruz sc-166574, 1:500), anti-β-Actin mouse monoclonal IgG (Santa Cruz sc-81178, 1:500), Goat anti-rabbit IgG-HRP (Santa Cruz sc-200416, 1:2,500), Rabbit anti-mouse IgG-HRP (Santa Cruz sc358917, 1:2,500). Full images of western blots are presented in Supplementary Fig. 10.

### RhoA activation assay

Pre-osteoclasts (derived from BMMs with 2-day treatment of RANKL and M-CSF) were starved for 4 h and stimulated by RANKL and M-CSF for different times. Cell lysates was harvested and RhoA-GTP activity was analysed using commercial kits (Rho Activation Assay Biochem Kit, Cytoskeleton, Inc.) according to the manufacturer's instructions.

### Constructs and virus production and infection

TRC lentiviral mouse *Gna13* targeted short hairpin RNA (Clone ID: TRCN0000098146, target sequence 5′-CGCGATCAACACAGAGAACAT-3′), TRC Lentiviral pLKO.1 Empty Vector and mouse *Gna13* full-length cDNA were purchased from Open Biosystems, Thermo Fisher Scientific Inc. Lentiviral pLB vector and packaging plasmids (pCMV-VSV-G and pCMV-Dr8.92) were purchased from Addgene. pHR-EF-IRES-Bla (referred to as pHR in the following text) was a gift from Dr Ling Tian in Dr W. Timothy Garvey's lab and used for lentivirus-mediated overexpression^22^. Gα13 constitutively active form (Gα13 Q226L, referred to as Gα13CA in the text) full-length cDNA was purchased from Missouri cDNA Resource Center. Akt constitutively active form cDNA was purchased from addgene. Gα13CA was ligated into pHR-EF-IRES-Bla at the *BamHI* and *XhoI* sites and substituted the green fluorescent protein fragments, to construct pHR.Gα13CA vector. Retrovirus vector pMXs-ires-puro was a gift from Dr Xu Feng. A 3xFLAG sequence was inserted into pMXs-ires-puro to construct pMXs-3xFLAG-ires-puro vector. Gα13CA was ligated into pMXs-3xFLAG-ires-puro at the EcoRI and NotI sites to construct pMXs-3xFLAG-G13CA-ires-puro vector (referred to as pMXs-Gα13CA). AktCA was ligated into pMXs-ires-puro vector to construct pMXs-AktCA vector. For lentivirus production, lentivirus vectors (with 10% pLB vector or 10% pHR vector) and packaging plasmids were co-transfected into HEK-293T cells using a calcium phosphate co-precipitation method. The lentiviral supernatant was harvested 48–72 h post-transfection. For retrovirus production, pMXs vectors was transfected into 293GPG cells (gift from Dr Xu Feng) using a calcium phosphate co-precipitation method and retrovirus supernatant were harvested between 48–96 h (ref. 46). Gα13CA was ligated into pAAV-CMV vector from Invitrogen at EcoRI and SalI sites to produce pAAV-CMV-G13CA vector (referred to as pAAV-G13CA). Lentivirus and retrovirus titers were determined by transfecting HEK293T cells with serial dilutions of virus supernatant. AAV was produced and tittered according to the manufacturer's instructions (Life Technology, Inc). BMMs or RAW264.7 cells were transduced with virus supernatant in the presence of 8 μg ml^−1^ polybrene (Sigma) for 24 h before induced with RANKL.

### OVX-induced bone destruction and AAV-G13CA treatment

OVX or sham was performed on two-month-old female mice. One week later and two weeks later those mice were administered with a local calvarial injection of 30 ul AAV (titer=10^9-10^ ml^−1^) expressing YFP or G13CA. Mice were harvested 5 weeks after OVX operation and fixed in 4% PFA. Calvaria bone were analysed by X-ray, u-CT and whole-mount TRAP staining. Calvarial were also decalcified for 3 days, immersed in 30% sucrose overnight and then submitted to frozen section and TRAP staining and immunofluorescent staining.

### Immunofluorescent staining

Frozen sections were fixed in 4% PFA for half an hour and heat-induced antigen retrieval was performed (abcam, ab973). Sections were incubated with mouse IgG blocking solution and universal blocking solution, and then overnight with primary antibodies: anti-YFP rabbit IgG (Santa Cruz sc-410, 1:100) or anti-Gα13 rabbit IgG (Santa Cruz sc-410, 1:100) and anti-Ctsk mouse IgG (Santa Cruz sc-48353, 1:200). Ctsk was then labelled by biotin-anti-mouse IgG (Vector laboratories, BMK-2202) and streptavidin-FITC (sigma). YFP and Gα13 were labelled by TR-goat-anti-rabbit IgG.

### LPS-induced bone destruction and AAV-G13CA treatment

Seven-week-old female mice were administered with a local calvarial injection of AAV expressing YFP or G13CA. Six days later those mice were administered with a local calvarial injection of LPS (sigma) at 25 mg kg^−1^ body weight or PBS and analysed after 5 days following a previously described model^25^. For analysis, calvarial with skin tissue were fixed in 4% PFA for 6 h at 4 °C, decalcified for 3 days, immersed in 30% sucrose overnight and then submitted to frozen section and TRAP staining.

### TNFα-induced rheumatoid arthritis and AAV-Gα13CA treatment

13-week old WT and human-TNFα-transgenic (hTNFtg) mice was injected with AAV (titer=10^9-10^ ml^−1^) expressing YFP or G13CA over ankles. The injection was performed four times every week successively. Photographic and radiographic analysis were performed before and 35 days after the first injection. Samples were harvested 35 days after the first injection, and Safranin O (SO) stain, H&E stain and TRAP stain were performed. Hind paw volume was quantified by water displacement method^47^. Bone destruction in X-ray was quantified by Larsen method in RA lesion area^48^. Cartilage destruction was assessed using safranin O staining and was measured as OARSI grade in ankle joint area^49^.

### Statistics

Data represent mean±s.d. Analysis was performed using GraphPad Prism, version 5. Statistical significance was assessed using a two-tailed Student's *t*-test or ANOVA analysis, as indicated in the figure legend, considering a *P* value≤0.05 as significant.

### Data availability

The authors declare that the data supporting the findings of this study are available within the article and its Supplementary Information files.

## Additional information

**How to cite this article:** Wu, M. *et al*. Gα13 negatively controls osteoclastogenesis through inhibition of the Akt-GSK3β-NFATc1 signalling pathway. *Nat. Commun*. **8**, 13700 doi: 10.1038/ncomms13700 (2017).

**Publisher's note:** Springer Nature remains neutral with regard to jurisdictional claims in published maps and institutional affiliations.

## Supplementary Material

Supplementary Information Supplementary Figures 1-10.
