# Author Correction: Gα13 negatively controls osteoclastogenesis through inhibition of the Akt-GSK3β-NFATc1 signalling pathway

**DOI:** 10.1038/s41467-019-13015-6

**Published:** 2019-11-25

**Authors:** Mengrui Wu, Wei Chen, Yun Lu, Guochun Zhu, Liang Hao, Yi-Ping Li

**Affiliations:** Department of Pathology, University of Alabama at Birmingham, SHEL 810, 1825 University Boulevard, Birmingham, AL 35294-2182 USA

Correction to: *Nature Communications,* 10.1038/ncomms13700; published online 19 January 2017.

In this Article, there are errors in the presentation of western blots experiments affecting the different controls (GAPDH, tubulin, β-actin) in Figs. 2b,  5i, Supplementary Figs. 1d, 6d, e. In Supplementary Fig. 1d the Western blot bands of different days were inadvertently switched, and there is unrelated raw data in Supplementary Fig. 10. However, the raw data of all the control bands were correctly labeled in Supplementary Fig. 10 and the raw data for Supplementary Fig. 1d were correctly labeled in Supplementary Fig. 10. In detail, in Fig. 2b, the endogenous control lanes in the right panel were inadvertently mislabeled as GAPDH rather than β-actin. In Fig. 5i and Supplementary Fig. 6d, and its quantification in Supplementary Fig. 6e, the endogenous control lanes were inadvertently mislabeled as GAPDH rather than tubulin. In Supplementary Fig. 1d, Day 2 β-actin bands were inadvertently switched with Day 3 β-actin bands, and Day 5 β-actin bands were inadvertently used for Day 4 β-actin bands. The three leftmost bands in the raw data for Fig. 4m in Supplementary Fig. 10 are not for Fig. 4m or any other data in this article, and the IκB and p-IκB bands in the raw data for Supplementary Fig. 7b in Supplementary Fig. 10 are not for Supplementary Fig. 7b or any other data in this article. The corrected versions of Figs. 2, 5, Supplementary Figs. 1, 6, and 10 appear below as correction Figs. 1–5, respectively. The corrections do not affect our original scientific findings and conclusions. The errors have not been corrected in the PDF or HTML versions of the Article.

**Fig. 1:**
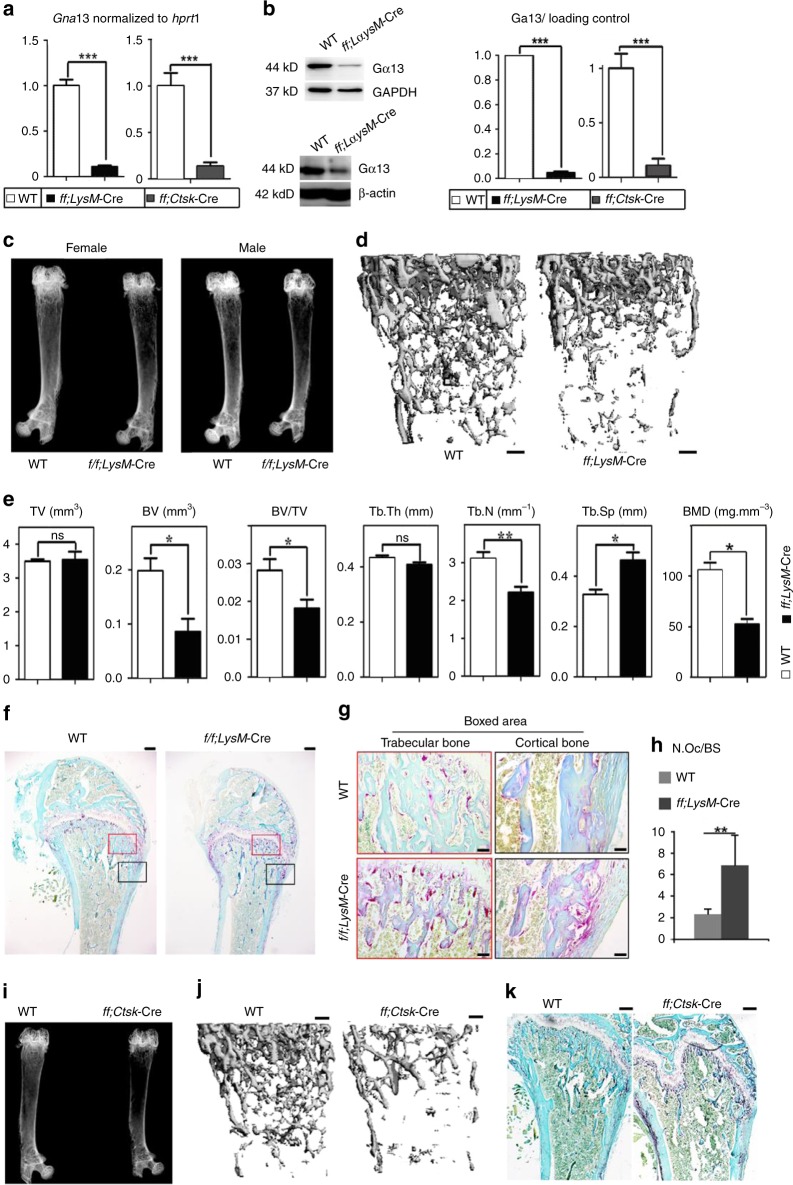
Osteoclast-specific Gna13-CKO mice displayed osteoporosis

**Fig. 2:**
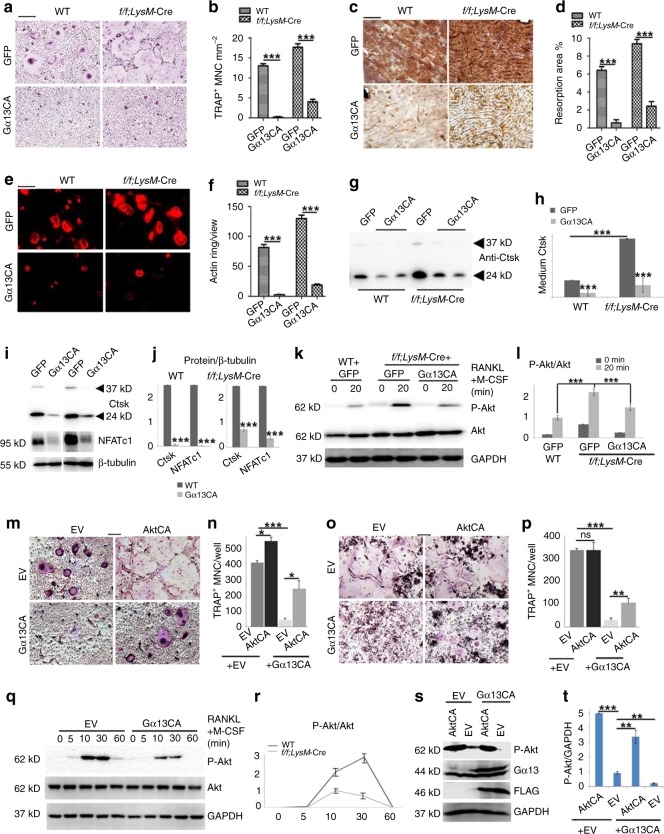
Ga13 gain-of-function inhibits osteoclast formation and function in vitro

**Fig. 3:**
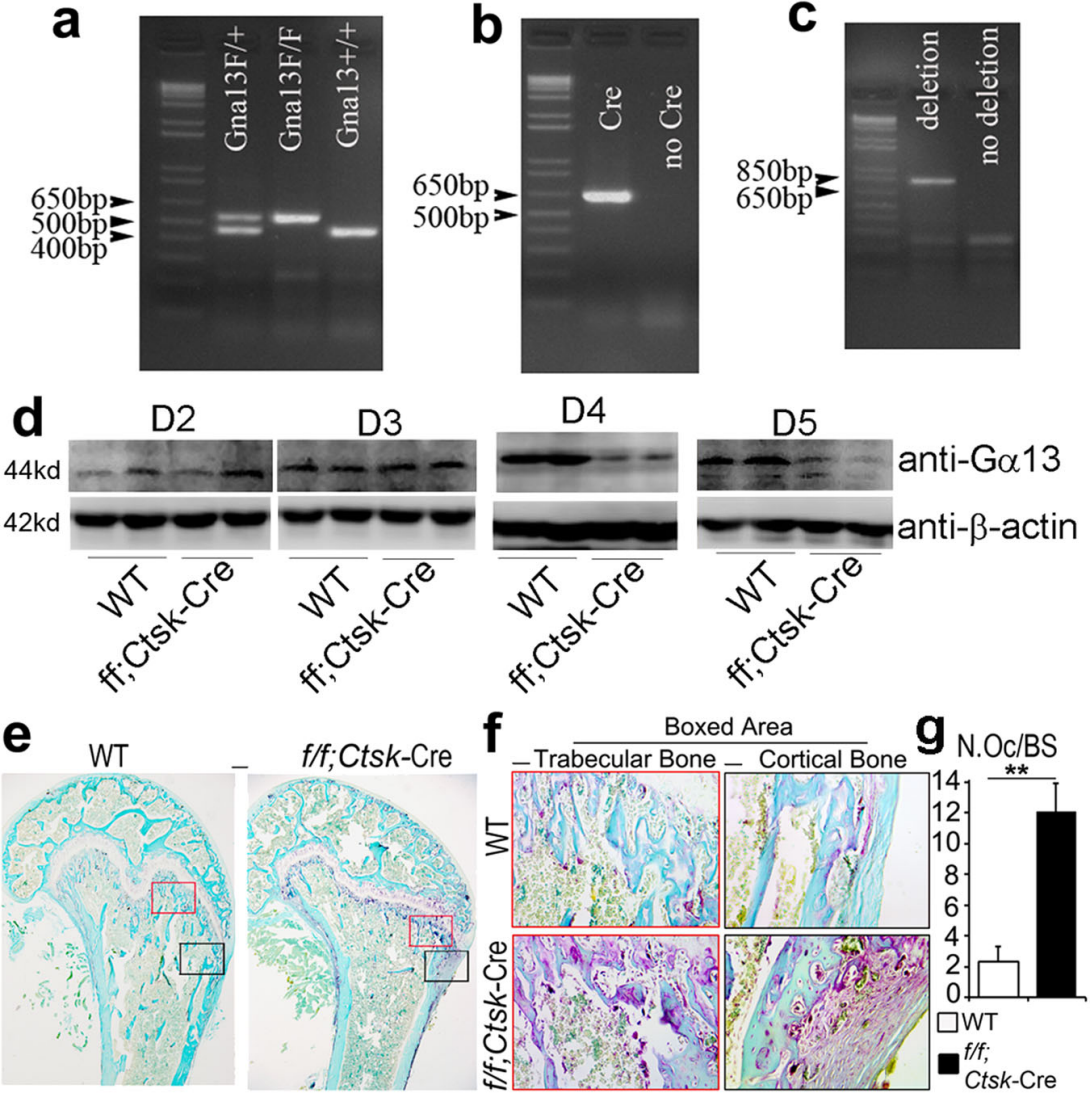
Bone density was decreased in osteoclast-lineage cell specific Gna13 deficient mice

**Fig. 4:**
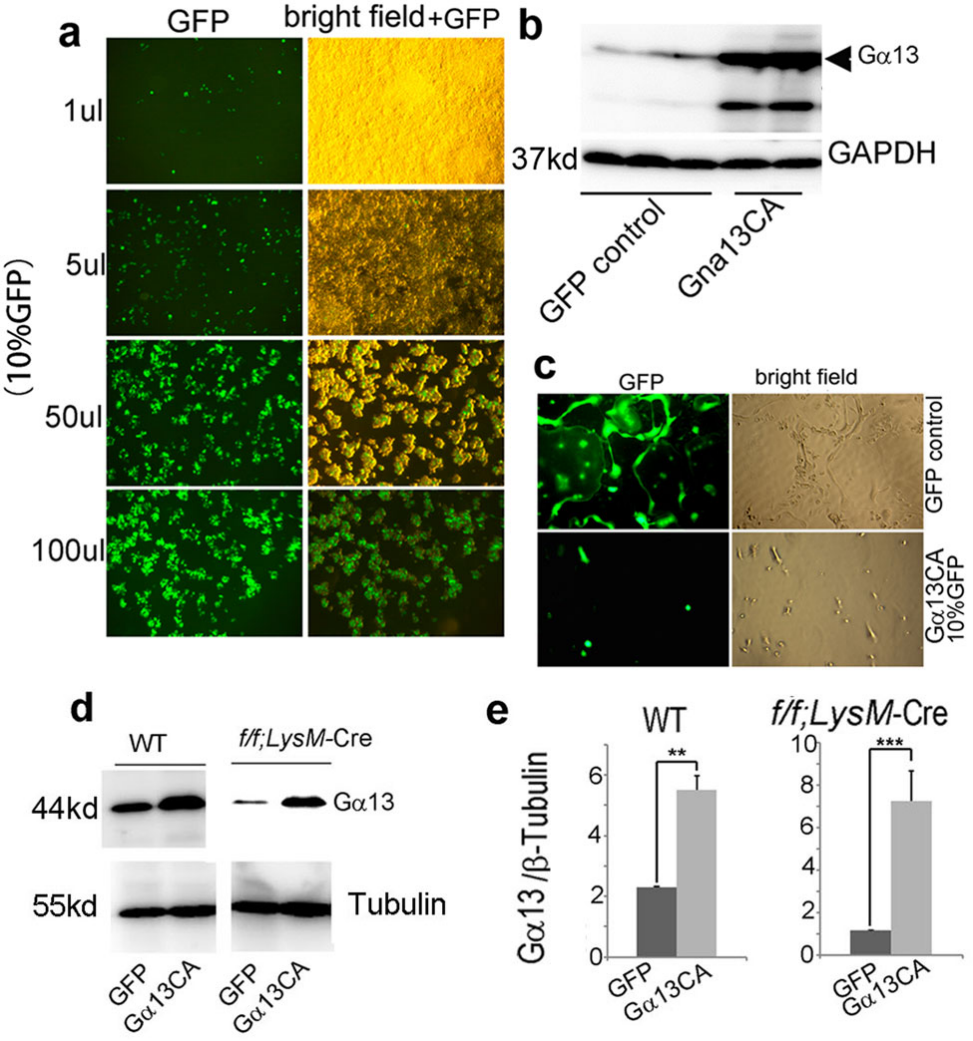
Analysis of the titer and expression of Gα13 overexpression lentivirus

**Fig. 5:**
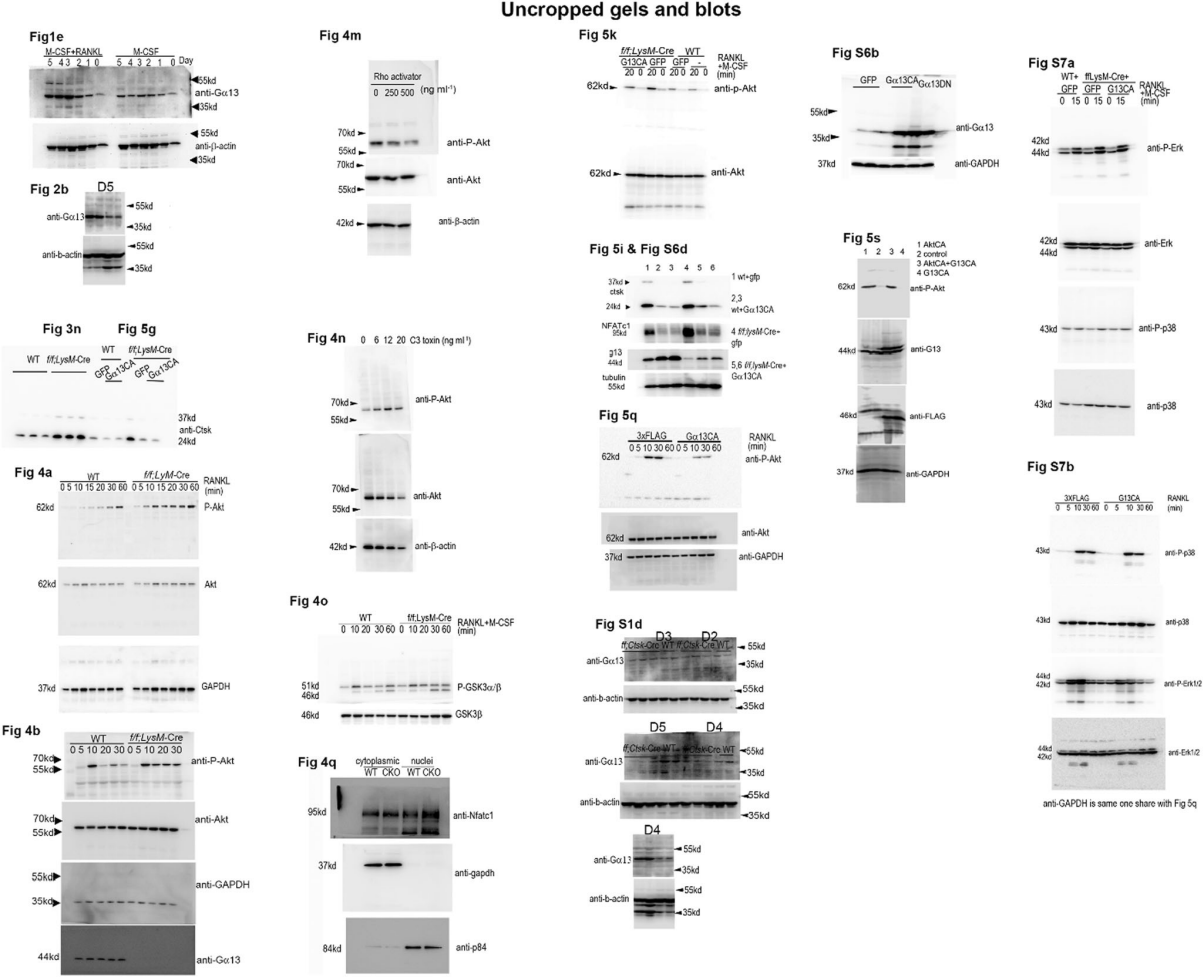
Uncropped images for western blots

