# Supplementary material for: Gα13 negatively controls osteoclastogenesis through inhibition of the Akt-GSK3β-NFATc1 signalling pathway
Source: Nat Commun. 2017 Jan 19;8:13700. doi: 10.1038/ncomms13700 (PMC5253683; doi:10.1038/ncomms13700)
Supplement: Supplementary Information — Supplementary Figures 1-10. [file ncomms13700-s1.pdf]

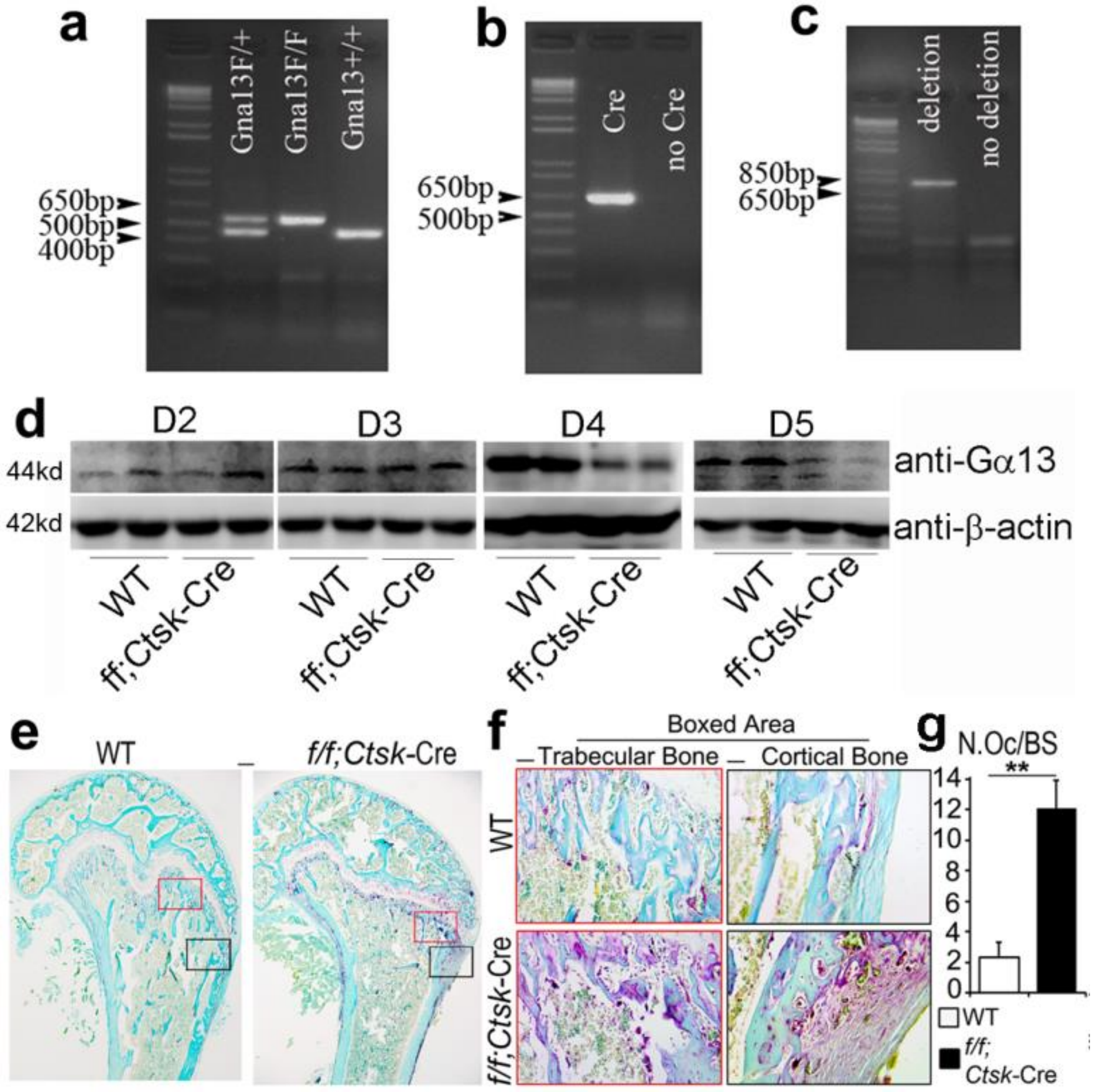

Supplementary Figure 1. Bone density was decreased in osteoclast-lineage cell specific *Gna13* deficient mice. (a-c) PCR genotyping of mice by mouse tail DNA. Primers were designed to detect *Gna13-WT/f* (~400bp/470bp) (a), *Gna13-deletion* (~800bp) (b) and *Cre* (~650bp) (c). (d) Gα13 protein levels in WT and *Gna13*$^{f/f}$*Cstk*-Cre (*f/f;Cstk*-Cre) bone marrow cells; D#, # days under the treatment of RANKL and M-CSF. (e,f) TRAP staining of femurs from two-month-old WT and *Gna13*$^{f/f}$*Cstk*-Cre female mice. (g) Quantification of osteoclast number in d. OcS/BS, osteoclast surface/bone surface. N.Oc/BS, osteoclast number/bone surface. Results were expressed as mean ± standard deviation (SD), N=6; **, p ≤ 0.01 (Student's T-test). scale bar in e, 200μm; scale bar in f, 20μm.

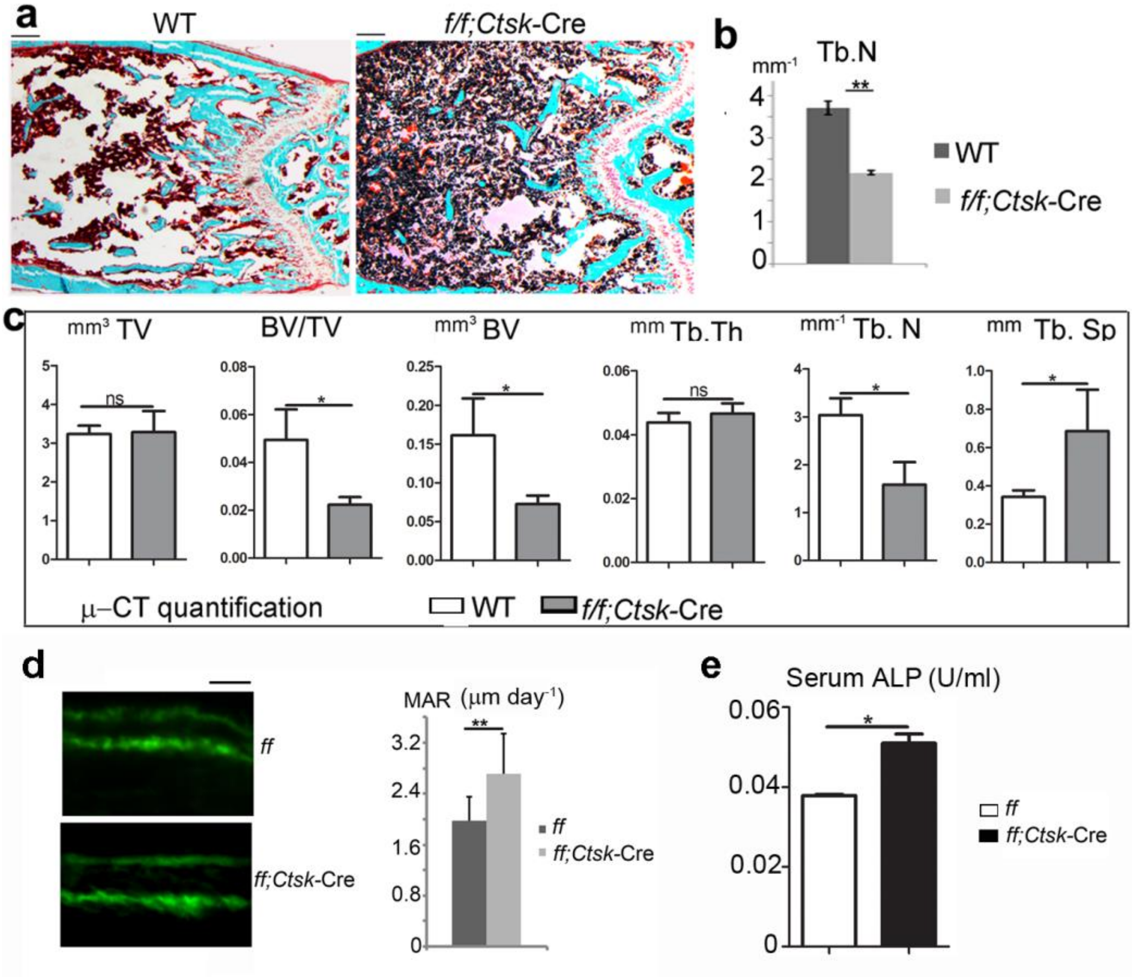

Supplementary Figure 2. Osteoclast number was increased in osteoclast-specific Gna13-deficient mice. (a) Trichrome staining of femurs from two-month-old female WT and *Gna13*$^{f/f}$*Cstk*-Cre (*f/f;Ctsk*-Cre) mice. (b) Histomorphometry quantification in a. ObS / BS, osteoblast surface / bone surface; N.Ob / BS, number of osteoblast / bone surface; Tb.N, trabecular bone number. (c) Quantification from μ-CT in Fig. 2i; N=4 Tb.Th, trabecular bone thickness; Tb.N, trabecular bone number; Tb.Sp, trabecular bone space; BV, bone volume; TV, tissue volume. (d) Calcein labeling in the 2-month-old female calvariae assessed using undecalcified frozen sections, and its quantification (MAR, mineral apposition rate); N=20. (g) Elisa to detect serum Alkaline Phosphatase (ALP) in 2-month old WT and *Gna13*$^{f/f}$*Cstk*-Cre female mice; N=4. Results were expressed as mean ± standard deviation (SD), N=3; *, p≤ 0.05; **, p ≤ 0.01; ns, not significant (Student's T-test). scale bar in a, 200μm; scale bar in d, 20μm.

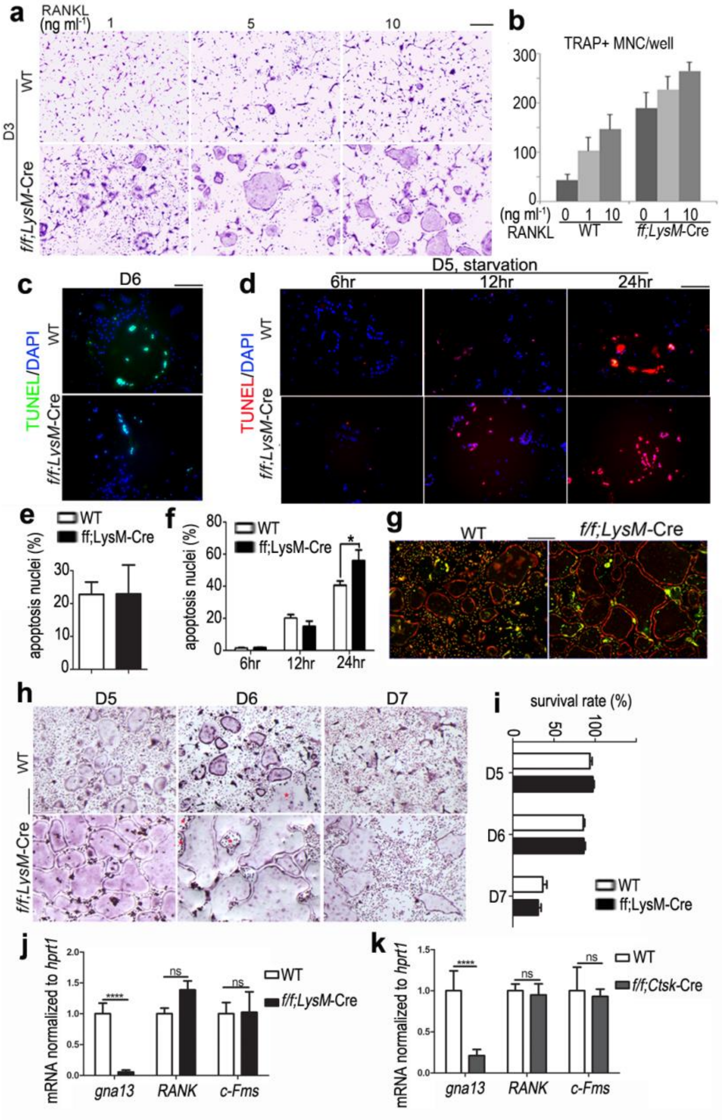

Supplementary Figure 3. Deletion of *Gna13* expression promotes osteoclast formation, but not survival and acidification.

(a) TRAP staining to detect osteoclast formation of WT and *Gna13*$^{f/f}$*LysM*-Cre (*f/f;LysM*-Cre) bone marrow monocytes (BMMs) under the treatment of M-CSF and different doses of RANKL for 3 days (D3). (b) Quantification of TRAP+ MNC per well in a; N=4 (c) TUENL staining of D6 WT and *Gna13*$^{f/f}$*LysM*-Cre osteoclasts. (d) TUENL staining of D5 WT and *Gna13*$^{f/f}$*LysM*-Cre osteoclasts, serum and cytokine starved for 6, 12 and 24 hours (hr). (e,f) quantification of apoptosis rate in c (e) and d (f); N=4 (g) Acidification of osteoclasts by acridine orange staining. (h) TRAP staining of extensive cultured WT and *Gna13*$^{f/f}$*LysM*-Cre osteoclasts. (i) Quantification of survival rated in h; N=4. (j) qRT-PCR to detect *gna13*, *RANK* and *c-Fms* expression in D1 WT and *Gna13*$^{f/f}$*LysM*-Cre pre-osteoclasts; N=4. (k) qRT-PCR to detect *gna13*, *RANK* and *c-Fms* expression in D4 WT and *Gna13*$^{f/f}$*Ctsk*-Cre osteoclasts; N=4. Results were expressed as mean ± standard deviation (SD); *, $p \leqslant 0.05$; **, $p \leqslant 0.01$; ***, $p \leqslant 0.001$; ****, $p \leqslant 0.0001$ (Student's T-test). scale bar in a,g,h 200μm; scale bar in c,d, 100μm.

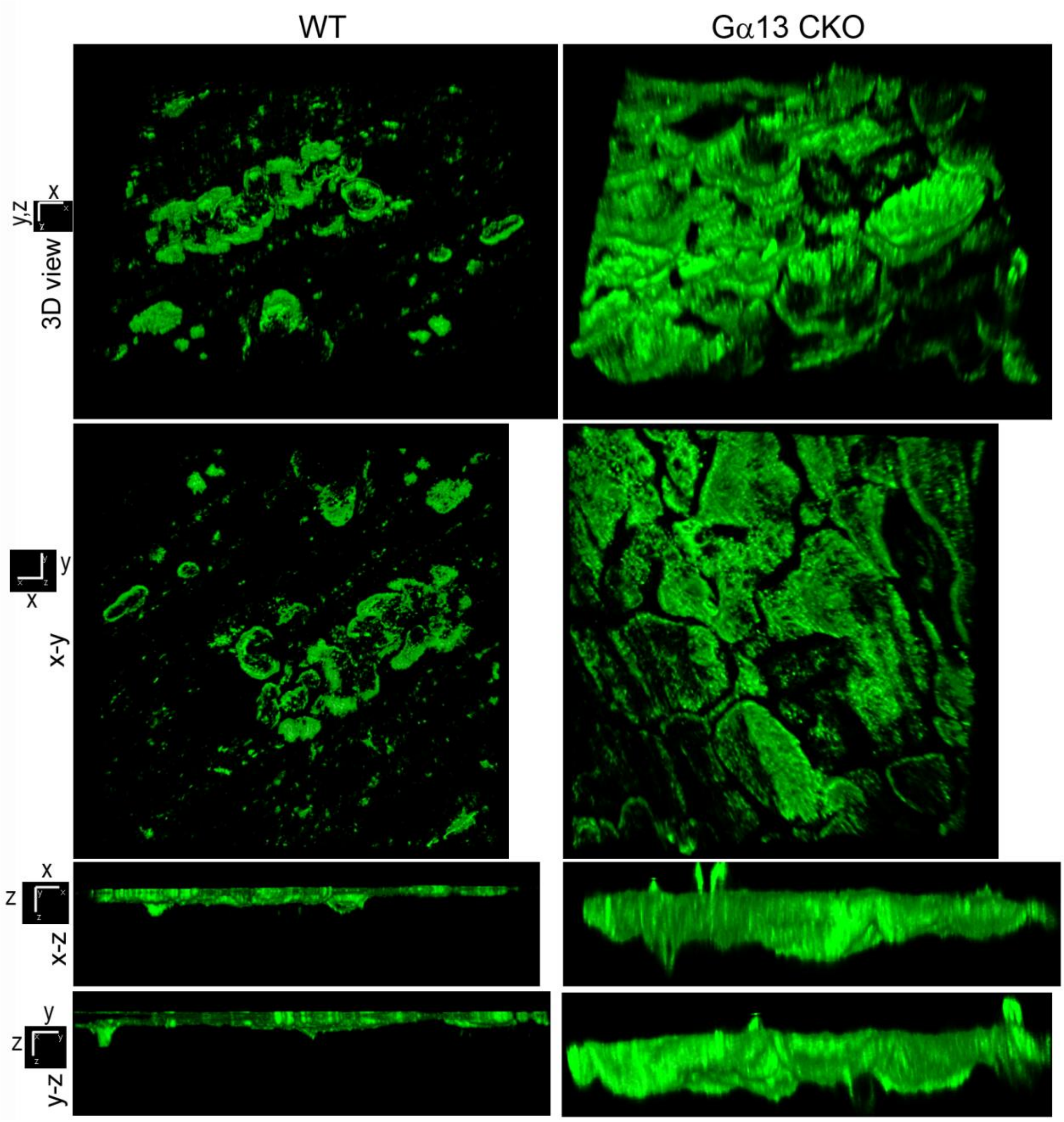

Supplementary Figure 4. Confocal microscopy of bone resorption pits resorbed by WT and *Gna13*$^{f/f}$*LysM*-Cre osteoclasts. scale bars: x, 0.13μm; y, 0.13μm; z, 0.45μm

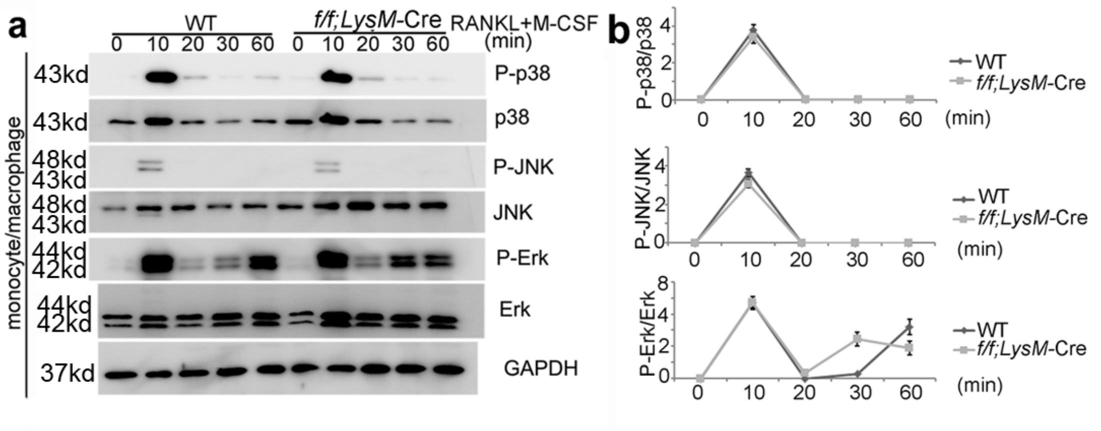

Supplementary Figure 5. p38, JNK and Erk phosphorylation is similar in WT and mutant cells. (a) Western blot analysis to detect phosphorylation signaling induced by RANKL and M-CSF in WT and *Gna13*$^{f/f}$*LysM*-Cre (*f/f;LysM*-Cre) BMMs. (b) Quantification of a by ImageJ. Results were expressed as mean ± standard deviation (SD), N≥3.

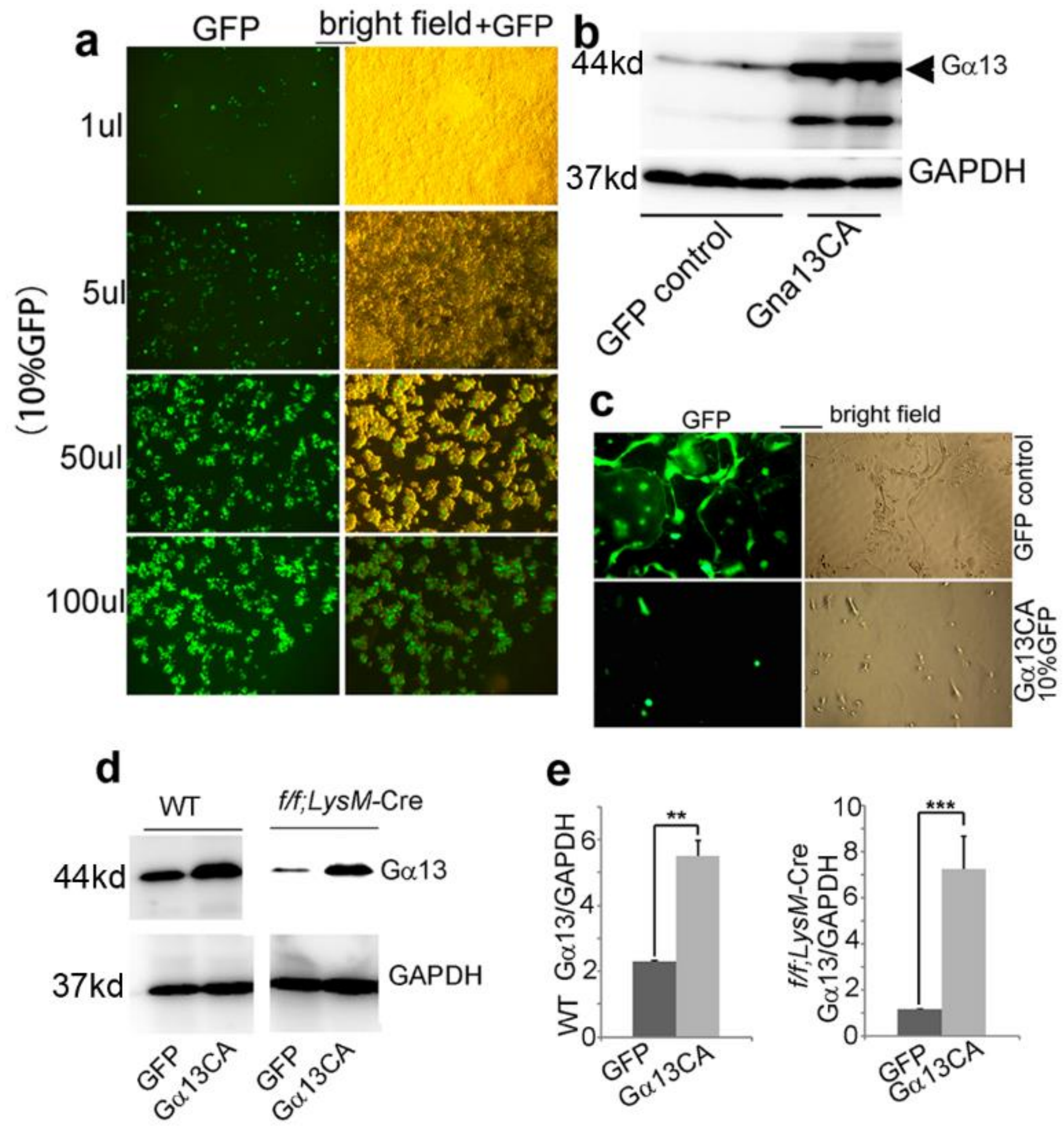

Supplementary Figure 6. Analysis of the titer and expression of Gα13 overexpression lentivirus.

(a) 293T was transfected with different volumes of Gα13 overexpressing lentivirus (containing 10% GFP expressing retrovirus), and GFP was observed 48 hours post-transfection. (b) Expression of Gα13 constitutive active form (Gα13CA) in 293T was confirmed by western blot. (c) Osteoclast precursors was transfected with GFP and Gα13CA overexpression lentivirus (the latter one contained 10% GFP expressing virus), and GFP was observed in mature cells. (d) WT and *Gna13*-deficient D1 pre-osteoclasts were transfected with GFP and Gα13CA overexpressing retrovirus and Gα13 expression was detected on Day 3 by western blot. (e) Quantification of d by ImageJ. Results were expressed as mean ± standard deviation (SD), N≥3; **, $p \leqslant 0.01$; ***, $p \leqslant 0.001$ (Student's T-test). Scale bar, 100μm

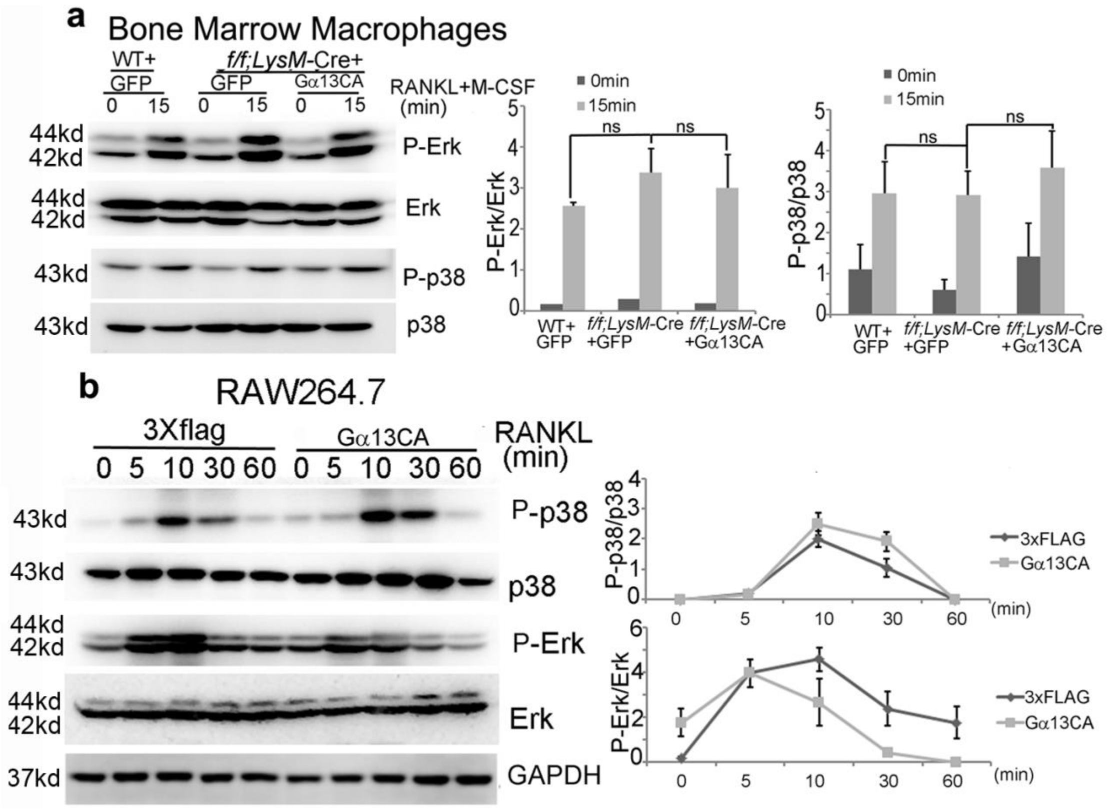

Supplementary Figure 7. Overexpression of Gα13CA does not affect p38 and Erk phosphorylation. (a) Western blot analysis to detect p38 and Erk phosphorylation induced by RANKL and M-CSF in WT and *Gna13*$^{f/f}$*LysM*-Cre (*f/f;LysM*-Cre) osteoclasts (overexpressing GFP or Gα13CA). (b) Western blot analysis to detect RANKL induced p38 and Erk phosphorylation in RAW264.7 cells transfected with control retrovirus and retrovirus expressing Gα13CA. Quantification data were co-presented on the right panel. Results were expressed as mean ± standard deviation (SD), N≥3; ns, not significant (two-way ANOVA analysis).

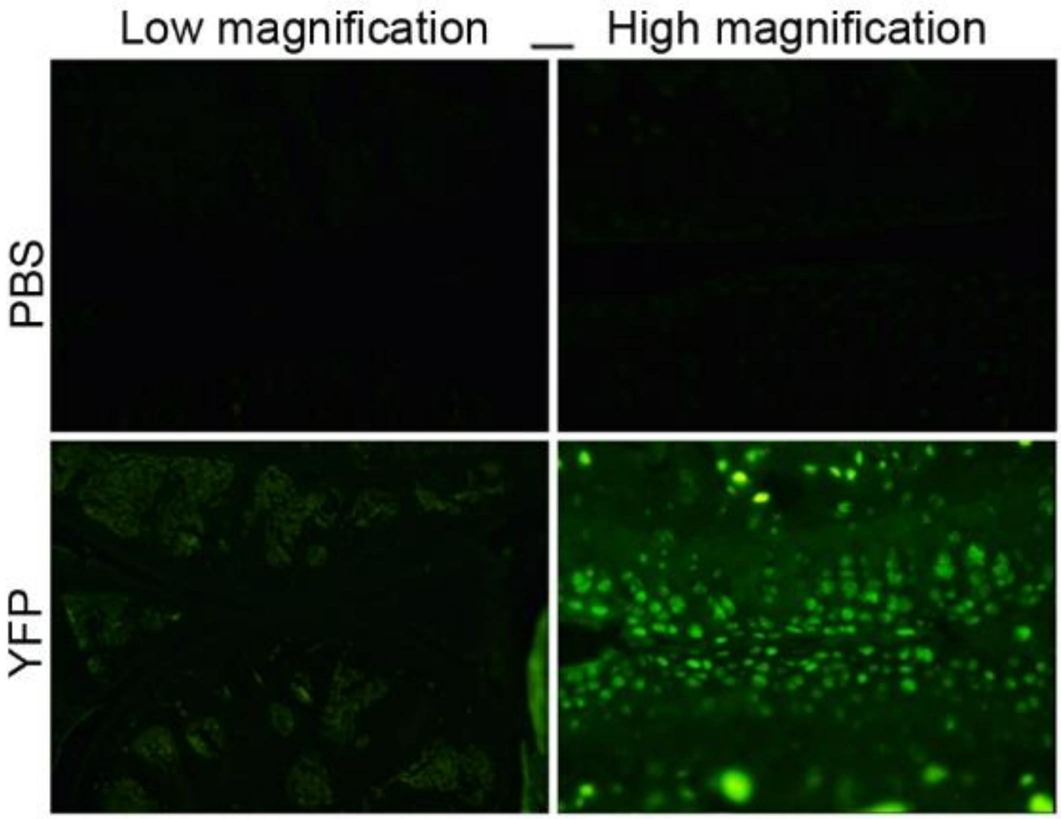

Supplementary Figure 8. Analysis of the effective infection of AAV in the knee joint. Upper panels, injection of PBS injection in knee Joint as control; Lower panels, injection of AAV-YFP in knee Joint, YFP expresses in the articular surface and Synovial cavity. Scale bar, 20μm

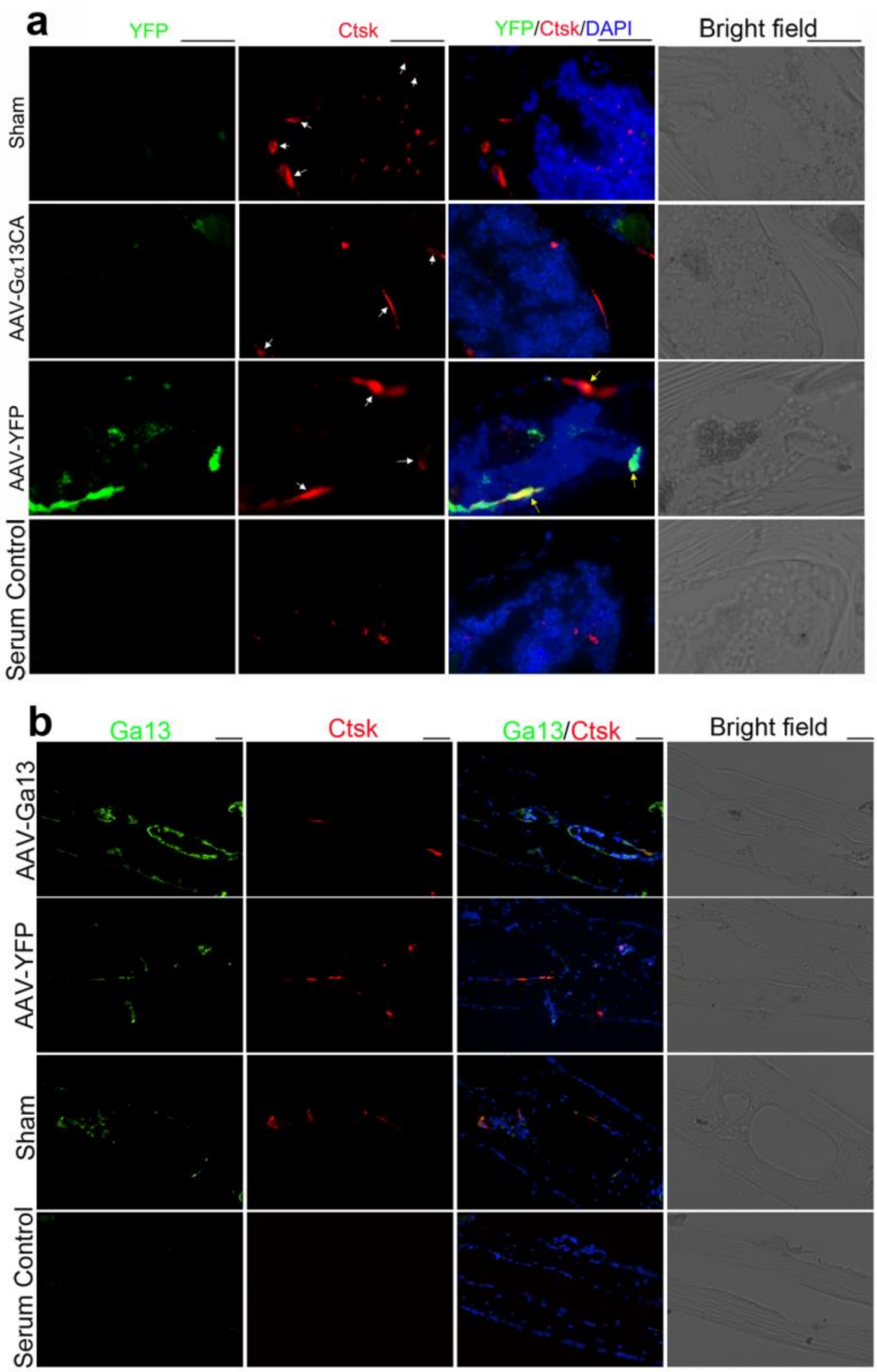

Supplementary Figure 9. Analysis of the effective infection of AAV in the 2-month female mouse calvarial bone in the ovariectomized (OVX) model. (a) Co-immunofluorescent staining using anti-YFP and anti-Ctsk antibody using frozen sections of calvarial bones. (b) Co-immunofluorescent staining using anti-Gα13 and anti-Ctsk antibody of calvarial bones. Scale bars, 100μm.

Uncropped gels and blots

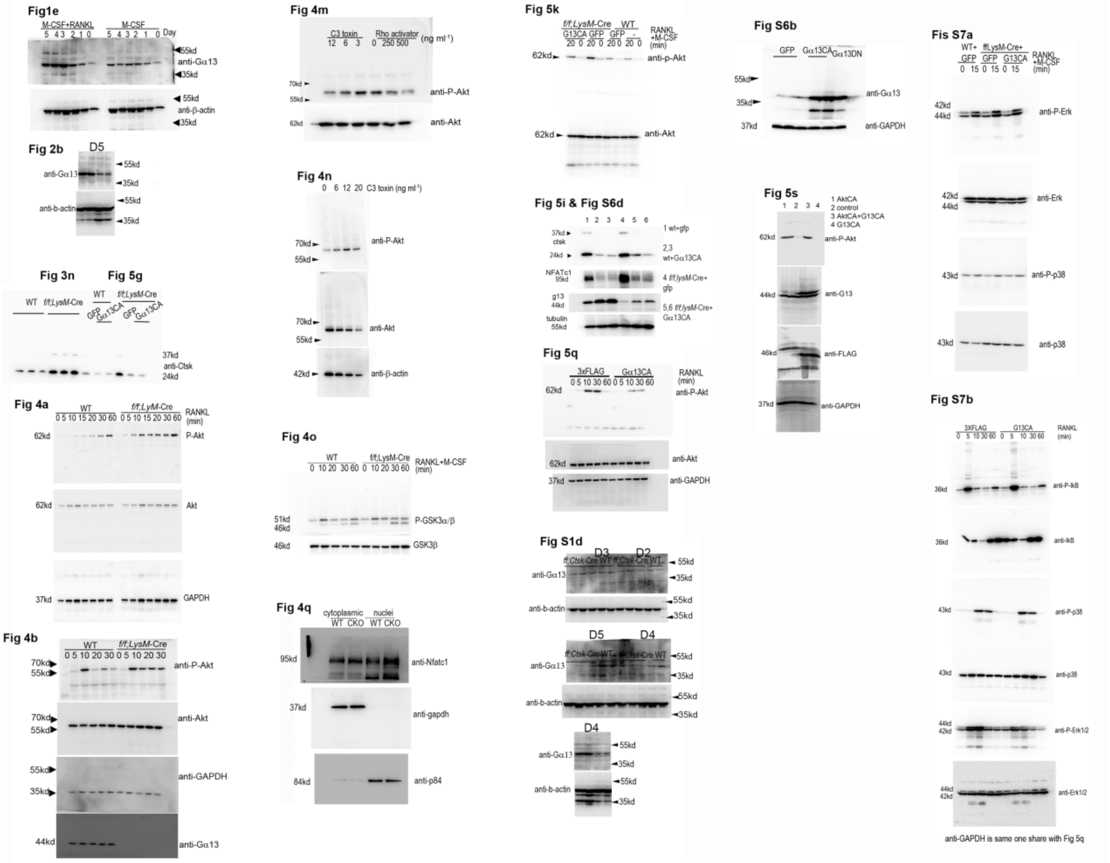

Supplementary Figure 10. Uncropped images for western blots
